# Hearing modulation affects Alzheimer’s disease progression linked to brain inflammation: a study in mouse models

**DOI:** 10.1186/s10020-024-01040-1

**Published:** 2024-12-26

**Authors:** Yoo-Seung Ko, Young-Kyoung Ryu, Sujin Han, Hyung Joon Park, Munyoung Choi, Byeong C. Kim, Han-Seong Jeong, Sujeong Jang, Jihoon Jo, Sungsu Lee, Won-Seok Choi, Hyong-Ho Cho

**Affiliations:** 1 Department of Otolaryngology-Head and Neck Surgery, Chonnam National University Medical School and Chonnam National University Hospital, 42 Jaebong-Ro, Dong-Gu, Gwangju, 61469 Republic of Korea; 2 Department of Biochemistry, University of Washington, 1959 NE Pacific Street, Seattle, WA 98195 USA; 3 Department of Neurology, Chonnam National University Medical School & Hospital, Gwangju, 61469 Republic of Korea; 4 Department of Physiology, Chonnam National University Medical School, Hwasun-Gun, Jeollanamdo, 58128 Republic of Korea; 5 School of Biological Sciences and Technology, Chonnam National University, Gwangju, Republic of Korea; 6 Department of Biomedical Sciences, Chonnam National University Medical School, Gwangju, Republic of Korea

**Keywords:** Drug-induced hearing loss, Alzheimer’s disease, Iba1, Glial fibrillary acidic protein, Neuroinflammation

## Abstract

**Background:**

Recent studies have identified hearing loss (HL) as a primary risk factor for Alzheimer’s disease (AD) onset. However, the mechanisms linking HL to AD are not fully understood. This study explored the effects of drug-induced hearing loss (DIHL) on the expression of proteins associated with AD progression in mouse models.

**Methods:**

DIHL was induced in 5xFAD and Tg2576 mice aged 3 to 3.5 weeks using kanamycin (700 mg/kg, subcutaneous) and furosemide (600 mg/kg, intraperitoneal). The accumulation and expression of beta-amyloid (Aβ), ionized calcium-binding adaptor molecule 1 (Iba1), and glial fibrillary acidic protein (GFAP) were measured through immunohistochemistry and immunoblotting. Additionally, the expression of proteins involved in the mammalian target of rapamycin (mTOR) pathway, including downstream effectors p70 ribosomal S6 kinase (p70S6K) and S6, as well as proinflammatory cytokines, was analyzed.

**Results:**

Compared to control conditions, HL led to a significant increase in the accumulation of Aβ in the hippocampus and cortex. Elevated levels of neuroinflammatory markers, including Iba1 and GFAP, as well as proinflammatory cytokines such as interleukin-1β (IL-1β), IL-6, and tumor necrosis factor-alpha (TNF-α), were observed. Moreover, DIHL enhanced phosphorylation of mTOR, p70S6K, and S6, indicating activation of the mTOR pathway.

**Conclusions:**

HL significantly increases Aβ accumulation in the brain. Furthermore, HL activates astrocytes and microglia, leading to increased neuroinflammation and thereby accelerating AD progression. These findings strongly suggest that HL contributes autonomously to neuroinflammation, highlighting the potential for early intervention in HL to reduce AD risk.

**Supplementary Information:**

The online version contains supplementary material available at 10.1186/s10020-024-01040-1.

## Introduction

Alzheimer’s disease (AD) is the leading cause of dementia worldwide. The Global Burden of Disease (GBD) study, a comprehensive research initiative assessing the worldwide effect of various health conditions, reports 57.4 million dementia cases in 2019, with estimates suggesting this will rise to 152.8 million by 2050 (Collaborators 2019, 2020). The Lancet Commissions have identified 12 potentially modifiable risk factors for dementia, including limited education, hypertension, hearing impairment, smoking, obesity, depression, physical inactivity, diabetes, social isolation, excessive alcohol consumption, head injuries, and air pollution (Livingston et al. 2020). Among these, hearing impairment presents the highest population fraction, with substantial evidence linking it to an increased risk of cognitive decline and dementia (Livingston et al. 2020). AD is characterized by amyloid plaques and neurofibrillary tangles (NFTs), which drive neurodegeneration and neuronal loss (Shen et al. 2021). The hereditary form of AD is connected to mutations in the amyloid precursor protein (APP) and presenilin 1 and 2 (PSEN1, PSEN2). These mutations disrupt normal APP processing, resulting in the accumulation of plaques and the development of early-onset familial Alzheimer's disease (Lane et al. 2018; Lanoiselee et al. 2017). In contrast, most AD cases are sporadic, with late-onset and a multifactorial etiology involving genetic and environmental factors. Genetic factors are estimated to contribute to approximately 70% of sporadic AD cases, with the ApoE gene being the most significant (Lane et al. 2018). Additionally, APP and PSEN mutations may influence sporadic AD by disrupting amyloid processing, resulting in the formation of toxic amyloid aggregates that drive neurodegeneration and disease progression (Lanoiselee et al. 2017). Furthermore, hearing impairment, a recognized modifiable risk factor for dementia (Livingston et al. 2020), could exacerbate AD progression. Hearing loss (HL) is associated with increased neuroinflammation (Shen et al. 2021) and amyloid deposition (Zheng et al. 2022), which may further worsen cognitive decline, linking APP, PSEN, and ApoE to familial and sporadic AD progression, particularly in the presence of HL.

According to the GBD study, 6–8% of the global population experiences disabling HL (Wilson et al. 2017). Furthermore, age-related HL has progressively risen in rank for years lived with disability, reaching fourth and seventh among men and women in the 2017 survey, respectively (Disease et al. 2018). With the aging global population, one-quarter of the population worldwide is projected to experience HL by 2050 (WHO 2021) **. The causes of HL are multifactorial, including genetic predisposition, infections, noise exposure, ototoxic drugs, trauma, immune responses, and aging. These factors primarily damage the outer hair cells, inner hair cells, spiral ganglion neurons, and stria vascularis in the cochlea (Wong and Ryan 2015). Extensive research exists on the intracellular signaling pathways involved in hair cell loss. Reactive oxygen species and reactive nitrogen species, induced by noise exposure and ototoxic drugs, play critical roles in hair cell damage (Henderson et al. 2006). In noise-induced models, MAPK phosphorylation and ERK1/2 signaling pathways are altered (Patel et al. 2013). Additionally, pro-inflammatory cytokines such as TNF-α, IL-1β, and IL-6 are activated by noise exposure and ototoxic drugs (Wakabayashi et al. 2010; Dinh C et al. 2013). A study also reports the roles of phosphatidylinositol 3-kinase, protein kinase C, and protein kinase B in hair cell loss and survival (Chung et al. 2006). Consequently, modulating these signaling pathways emerges as a potential therapeutic strategy for treating sensorineural HL (Wong and Ryan 2015).

As previously mentioned, a strong correlation is believed to exist between HL and dementia. A longitudinal study involving 639 participants was conducted. The results show that individuals with moderate HL have more than three times the risk of developing dementia than those with normal hearing. In contrast, those with severe HL have nearly a 4.94-fold higher risk (Lin et al. 2011a). Several mechanisms have been proposed to explain this association, including shared pathologies, reduced cognitive reserve due to an impoverished environment, increased cognitive load for listening, and interactions between auditory-related brain activity and dementia pathology (Griffiths et al. 2020). Although establishing a causal relationship in individuals is challenging, various animal studies provide insight. A study reports that noise-induced HL causes central nervous system inflammation, elevated glucocorticoid levels, oxidative stress, and increased excitotoxic glutamate in the hippocampus (Nadhimi and Llano 2021). However, the precise underlying mechanisms remain unclear, and studies on HL-related molecular changes that lead to cognitive decline are limited. Microglia and astrocytes, two glial cell types in the central nervous system, play critical roles in inflammation regulation and contribute significantly to AD pathology through their activation (Zhang and Jiang 2015).

Abnormal Aβ accumulation triggers an inflammatory response in AD, leading to cellular damage. Elevated levels of inflammatory mediators in brain tissue exacerbate nerve cell damage, contributing to cognitive decline (Weaver 2020). The inflammatory response in AD is a critical mechanism underlying neuronal damage and impaired cognitive function. Understanding the complex interactions between neurons, microglia, astrocytes, and other factors is vital for developing strategies to delay or prevent AD progression. Studies on the serine/threonine kinase mTOR, a regulator of cell survival and aging, report that mTOR inhibitors (such as rapamycin) may show neuroprotective effects in AD models. Rapamycin could delay AD progression by reducing Aβ deposition, preserving brain function in *APOE*ε4 gene carriers, alleviating chronic inflammation, and enhancing cognitive performance (Hou et al. 2023). mTOR plays a role in regulating pro-inflammatory and anti-inflammatory cytokine levels (Temiz-Resitoglu et al. 2017). Additionally, abnormal upregulation of mTOR signaling is observed in AD brains, particularly during neurodegeneration (Tramutola et al. 2015). Dysregulation of mTOR signaling is related to AD progression. Data from AD brains show increased phosphorylation of mTOR at Ser-2448 and elevated phosphorylation of its downstream target, p70S6K, in the hippocampus and cortex (Pei and Hugon 2008; Li et al. 2005). Moreover, mTOR hyperactivity correlates with the Braak stage and/or cognitive severity (Tramutola et al. 2015).

Therefore, this study aims to elucidate the molecular changes in the brain associated with auditory impairment and AD development and to investigate the mechanisms linking HL to AD progression. We employed kanamycin and furosemide (Kros et al. 2019; Selimoglu 2007; Ruggero and Rich 1991) to induce HL in 5xFAD mice, a genetically predisposed AD model. Subsequently, we examined the involvement of Aβ accumulation and neuroinflammation. This study could clarify how hearing impairment contributes to AD pathology at the molecular level, suggesting potential therapeutic targets for mitigating neurodegeneration in patients with AD.

## Materials and methods

### Animals

In this study, 5xFAD and Tg2576 transgenic mice served as models for AD. Wild-type (WT) mice, originating from the B6SJLF/J background, were obtained from the Jackson Laboratory (Bar Harbor, ME, USA). These mice had access to ample food and water. The animal experiments adhered strictly to the Guide for the Care and Use of Laboratory Animals of Chonnam National University. The protocol received approval from the Committee on the Ethics of Animal Experiments of Chonnam National University (CNUHIACUC-21048). Throughout the study, all the mice survived. For anesthesia, ketamine (80 mg/kg) and xylazine (10 mg/kg) were administered intraperitoneally.

### Drug administration

Kanamycin sulfate and furosemide were procured from Sigma‒Aldrich (K1377, St. Louis, MO, USA) and Dong-A Pharmaceutical Co., Ltd. (Chungbuk, Korea), respectively. A drug-induced hearing loss animal model was established in 3–3.5-week-old mice through a subcutaneous injection of kanamycin (700 mg/kg body weight), followed 20 min later by an intraperitoneal injection of furosemide (600 mg/kg body weight). To mitigate dehydration, the mice received an intraperitoneal injection of glucose (50 mg/kg body weight) from JW Life Science Corp. (Chungnam, Korea). Control mice were injected with PBS, and auditory brainstem response (ABR) measurements were taken following the administration of kanamycin and furosemide.

### Auditory brainstem response

One month after drug administration, was measured using click and tone burst stimuli in the left and right ears. Body temperature was maintained with a heat therapy pump (#TP700, MI, USA). All animals were anesthetized with an intraperitoneal injection of ketamine (80 mg/kg) and xylazine (10 mg/kg) before being placed in an audiometric booth. The ABR was recorded using a 3RZ6 TDT system (Tucker–Davis Technologies, Alachua, FL, USA), which delivered stimuli ranging from clicks to tone bursts. Subdermal needle electrodes (1.5 mm) were inserted at the dorsal midline between the eyes (noninverting), on the scalp, and posterior to both pinnae. For each frequency, stimuli were presented at intensity levels decreasing from 90–20 dB below the visual ABR threshold.

### Object recognition memory test

The experimental setup consisted of a white acrylic Plexiglas square open field with dimensions of 45 cm × 45 cm × 45 cm. Habituation training was conducted over 3 days, during which the animals were placed in the apparatus for 10 min daily without any objects. The training session occurred 24-h after the final habituation session. In this session, the mice were placed in the apparatus with two identical objects and allowed to explore for 10 min. Following a 24-h retention interval, the mice were reintroduced to the apparatus, where one of the original objects was replaced with a new one. During this session, the mice were allowed to explore for 5 min. The objects used in this experiment were plastic toys of similar height. The time the mice spent exploring each object (familiar object, *T*_*familiar*_; novel object, *T*_*novel*_) was recorded. The discrimination index was calculated as follows: (*T*_*novel*_ − *T*_*familiar*_)/(*T*_*novel*_ + *T*_*familiar*_) × 100.

### Y-maze spontaneous alternation

Mice underwent spontaneous alternation testing in a Y-maze constructed from white acrylic Plexiglas, consisting of three arms labeled A, B, and C. Each arm measured 40, 6.8, and 15.5 cm in length, width, and height, respectively, with a 120° angle at each junction. The mice were allowed to explore the maze independently for 5 min. During this time, we counted the instances where the tail of a mouse fully entered each arm and noted the sequence of arm entries (following an A, B, or C pattern). Each complete sequence of entries into all three arms earned one point (termed actual alternation). Between trials, the interior of the Y-maze was cleaned with 70% ethanol, followed by 0.5% acetic acid. Spontaneous alternation behavior, defined as entry into all three arms consecutively without repetition, was calculated using the following formula: rate of spontaneous alternation performance (%) = (number of alternations)/(total number of arm entries − 2) × 100. All tests were conducted by a technician blinded to the genotype of the animals.

### Mouse brain tissue isolation and preparation

The animals were euthanized via cervical dislocation, and the entire brain was promptly and carefully extracted, followed by sagittal bisection. The left hemisphere was separated into the hippocampus, striatum, cerebellum, and cortex. To isolate the hippocampus, one spatula was utilized to stabilize the cerebral cortex, while the tip of a second spatula was positioned beneath the ventral hippocampus. The hippocampus was then gently rolled out and detached from the cortex. The entire cortex was retained for Western blot analysis. Each brain region was homogenized in an ice bath using 1 × protein lysis buffer. The homogenates were centrifuged at 14,000 rpm for 15 min at 4 °C, and the resulting supernatants were collected for Western blotting. The right hemispheres were fixed in 4% paraformaldehyde (Duksan, Chungnam, Korea). Subsequently, the preserved brains were sectioned coronally at 40 μm using a vibratome (Vibratome VT1200S, Leica Microsystems GmbH, Germany).

### Electrophysiology

Hippocampal slices were placed in a recording chamber and continuously perfused with oxygenated aCSF at a flow rate of mL/min. The temperature was maintained at 29–30 °C. For recordings, two stimulating electrodes were placed on the Schaffer collateral pathway (to induced long-term potentiation (LTP) input) and subiculum region (for control input) to generate field excitatory postsynaptic potentials (fEPSPs). Recording glass pipettes were prepared using a micropipette puller (Sutter Instrument, P-1000) and filled with 3 M NaCl. The pipettes were fixed and controlled using a micromanipulator to position the recording electrode in the CA1 region of the hippocampus. After establishing a stable baseline for 30 min, two trains of tetanus stimuli (100 Hz for 1 s, with a 30 s intertetanus interval) were applied to induce LTP. Data acquisition and analysis were performed using WinLTP (www.winltp.com). The slope of evoked fEPSPs was measured, normalized to the preconditioning baseline, and expressed as a percentage of the baseline. Data were analyzed from one slice per mouse (n = number of slices = number of mice). In electrophysiology, the fEPSP slope is reported as the mean ± SEM (standard error of the mean) relative to the normalized baseline. To compare LTP between groups, fEPSPs recorded at the end of the experiment were analyzed.

### Immunofluorescence

The sections were washed three times with PBS containing 0.1% Triton X-100, blocked with 5% donkey serum for 1 h at room temperature, and incubated overnight at 4°C with the following primary antibodies: mouse anti-β-Amyloid (1:500, Santa Cruz #sc28365), mouse anti-AT8 (1:1000, Thermo #MN1208), rabbit anti-phospho-S6 (ser235/236, 1:500, CST #2211), rabbit anti-Iba1 (1:500, Wako #019-19741), and rabbit anti-GFAP (1:500, DAKO #Z0334). The sections were then incubated for 1 h at room temperature with Alexa Fluor 488-conjugated secondary antibodies: donkey anti-mouse (1:500, Jackson ImmunoResearch #715-545-150) or donkey anti-rabbit (1:500, Jackson ImmunoResearch #711-545-152). All sections were counterstained with DAPI (1:10000, Invitrogen, MP01306) for 10 min at room temperature to label the nuclei. Staining and visualization were conducted using an LSM 510-META confocal laser scanning microscope (Zeiss, Germany). For quantifying Aβ plaques, 10 × magnification images of β-amyloid staining were analyzed, and plaques larger than 50 μm^2^ in the cortex and hippocampus were counted using ImageJ (National Institutes of Health, Bethesda, MD, USA). The plaques were quantified and normalized to the unit area. We selected a plaque size threshold of 50 μm^2^, as this was the smallest area that distinguished between WT and 5xFAD mice. Phosphorylated S6 (pS6), Iba1, and GFAP-positive intensities in the cortex and hippocampus were measured using ZEN Microscopy Software (Zeiss, Germany). The results are presented as bar graphs (mean ± SEM, n = 7–8).

### Antibodies and western blotting

Aβ, IL-1β, IL-6, and TNF-α antibodies were obtained from Santa Cruz Biotechnology (Dallas, TX, USA). The pTau (AT8) antibody was procured from Thermo Fisher (Waltham, MA, USA), while Aβ, p-mTOR, mTOR, p-p70S6K, p70S6K, pERK, ERK, pS6, S6, and Tau antibodies were obtained from Cell Signaling Technology (Danvers, MA, USA). Actin and BACE1 antibodies were sourced from Sigma-Aldrich (St. Louis, MO, USA), along with appropriate secondary antibodies from MBL (Shirley, NY, USA). For Western blotting, proteins were separated via 10% or 12% polyacrylamide gel electrophoresis and transferred to polyvinylidene difluoride membranes. The membranes were incubated with primary antibodies diluted following the instructions of the manufacturer, at 4°C. Horseradish peroxidase-conjugated anti-mouse, or anti-rabbit IgGs were used as secondary antibodies. The blots were reprobed with an anti-actin antibody as a loading control. Immunoreactive proteins were then visualized using an enhanced chemiluminescence protocol. Protein levels were determined using densitometry, with relative protein levels normalized to those of actin. Data are presented as bar graphs (mean ± SEM, n = 5–8).

### Statistical analysis

Data are presented as the mean ± SEM. Statistical analyses were performed using GraphPad Prism 6.0 software (La Jolla, CA, USA). Differences among several groups were assessed using one-way analysis of variance (ANOVA), followed by Tukey’s post hoc test to identify specific group differences. For pairwise comparisons between two groups, unpaired two-tailed Student’s *t*-tests were applied. A *p*-value of < 0.05 was considered statistically significant. Significance levels are indicated as follows: ***P < 0.001; **P < 0.01, *P < 0.05.

## Results

### Alzheimer’s disease does not directly induce hearing loss

We selected the 5xFAD mouse model for its rapid and aggressive accumulation of Aβ plaques, which usually occurs within 4–6 months, making it ideal for studying early-stage AD (Spires-Jones and Knafo 2012). In contrast, Tg2576 mice were used for behavioral and LTP assessments, as they develop cognitive deficits around 12 months, coinciding with plaque buildup between 11 and 13 months (Irizarry et al. 1997). Although previous studies show a strong link between HL and AD (Gallacher et al. 2012; Lin et al. 2011b), our findings indicate that AD pathology did not result in hearing impairments in 6-month-old 5xFAD or 14-month-old Tg2576 mice (Supplementary Figure 1). Tone burst assessments revealed slight threshold shifts between 24 kHz and 32 kHz, but no significant differences were found at these frequencies between AD and WT mice. This suggests that HL may not be directly attributable to AD pathology alone. This finding aligns with that of studies indicating that cognitive decline in AD does not necessarily cause auditory dysfunction without additional factors such as induced HL (Lin et al. 2011b).

### Establishment of the mouse model of drug-induced hearing loss

To investigate the influence of HL on AD progression, we established a reliable model of HL in AD and WT mice. To induce HL, we administered a combination of kanamycin and furosemide, two well-known ototoxic drugs. Kanamycin leads to HL by causing cellular damage and hindering protein synthesis (Kros et al. 2019; Selimoglu 2007), whereas furosemide exacerbates HL by inhibiting diuretic enzymes through receptors in auditory cells (Ruggero and Rich 1991). Together, these drugs demonstrate synergistic effects, resulting in ototoxic injury and potentially causing permanent threshold shifts (Hirose and Sato 2011).

**Fig. 1:**
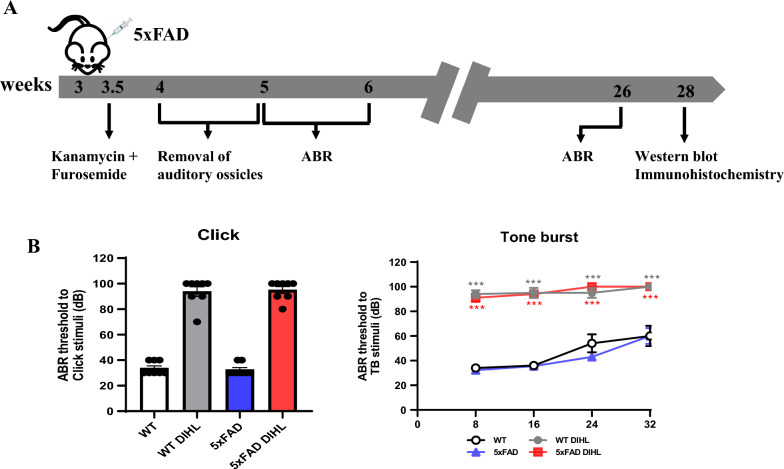
Establishment of a DIHL model induced by a combination of kanamycin and furosemide injections in 5xFAD mice. HL was induced in both WT and AD mouse models. At 3–3.5 weeks of age, the DIHL model was established by administering a subcutaneous injection of kanamycin at a dosage of 700 mg/kg body weight, followed 20 mins later by an intraperitoneal injection of furosemide at 600 mg/kg body weight. **A** Experimental timeline of 5xFAD mice. **B** ABR thresholds in 5xFAD mice were evaluated using click and tone burst stimuli (*n* = 8). Data are presented as the mean ± SEM. Statistical significance was assessed using ANOVA with Tukey’s post hoc test, supplemented by Student’s *t*-test where appropriate. Significance levels are indicated as *P < 0.05, **P < 0.01, and ***P < 0.001

To create the HL model, we administered subcutaneous and intraperitoneal injections of kanamycin at 700 mg/kg and furosemide at 600 mg/kg to 3–3.5 week-old mice, respectively. Prior to treatment, all mice had ABR thresholds < 30 dB SPL for test stimuli in both ears. Ossicle removal was performed between 4 and 5 weeks of age in the WT and 5xFAD mice to ensure the establishment of a deaf mouse model (Fig. 1A). ABR measurements were conducted at two time points (Lane et al. 2018; Lanoiselee et al. 2017; Li et al. 2005) to validate long-term HL.

At 26 weeks, the DIHL group exhibited significantly increased ABR thresholds (95 ± 3 dB SPL in the WT group; 94 ± 3 dB SPL in the 5xFAD group) compared to that of the controls (34 ± 3 dB SPL in the WT group; 32 ± 3 dB SPL in the 5xFAD group) (Fig. 1B). Additionally, tone burst ABR thresholds were significantly elevated at 8 (94 ± 3 dB SPL in the WT group; 91 ± 3 dB SPL in the 5xFAD group), 16 (95 ± 4 dB SPL in the WT group; 94 ± 2 dB SPL in the 5xFAD group), 24 (95 ± 4 dB SPL in the WT group; 100 ± 0 dB SPL in the 5xFAD group), and 32 kHz (100 ± 0 dB SPL in both groups), confirming complete HL (P < 0.001).

We also developed a HL model using Tg2576 mice (Supplementary Figure 2). Studies report a link between HL and cognitive decline or dementia (Lin 2011; Lin et al. 2013). In this study, the DIHL group exhibited substantial behavioral changes, including a significant decrease in interest in novel objects and spontaneous alternation performance, as well as an increase in same-arm returns compared to that of the control group (Supplementary Figure 3). These findings suggest that HL exacerbates cognitive dysfunction, supporting the hypothesis that HL contributes to cognitive decline.

To investigate the effect of HL on memory retention, we performed LTP experiments in the hippocampus, a region critical for memory and learning (Morrison and Baxter 2012; Bliss and Collingridge 1993). Supplementary Figure 4 shows that the WT and Tg2576 mice in the DIHL group exhibited a significant decline in LTP compared to that in the control group. These findings indicate that HL worsens cognitive dysfunction and is strongly associated with synaptic deficits in the hippocampus, ultimately impairing long-term memory. This highlights HL as a potential aggravating factor in AD progression AD.

### Hearing impairment enhances amyloid precursor protein expression and amyloid-beta accumulation in the brain tissue of 5xFAD mice

**Fig. 2:**
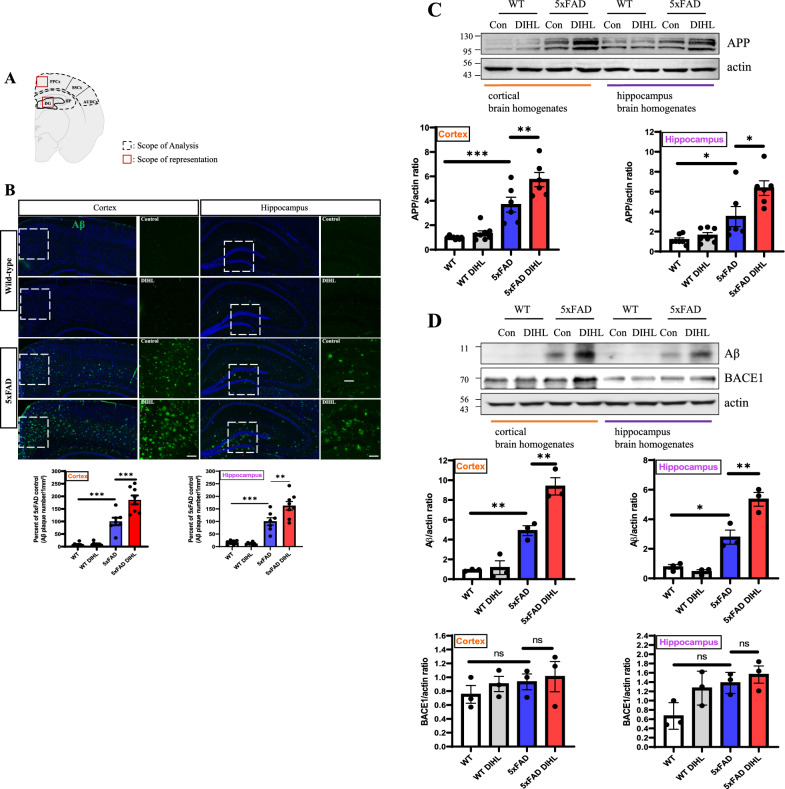
Hearing impairment significantly increases the expression of APP and the accumulation of Aβ in the brain tissue of 5xFAD mice. Brain regions for image acquisition were identified via immunohistochemical staining. Abbreviations: PPCx, posterior parietal cortex; SSCx, somatosensory cortex; AUDCx, auditory cortex; HP, hippocampus; DG, dentate gyrus (located within the hippocampus). The AUDCx was divided into dorsal, primary, and ventral regions, with only the dorsal and primary regions included in the analysis. Analyses of the specified regions and representative image locations were performed using images captured at 100 × magnification from the delineated areas at each location. **A** Analyzed brain regions and areas illustrated in the representative images. **B** Representative images showing Aβ plaque immunoreactivity in the cortex (left panel) and hippocampus (right panel) of WT and 5xFAD mice. Data are presented as the number of Aβ plaques per 1 mm^2^ (*n* = 7–8). Scale bars indicate 100 μm. **C** Western blot analysis of APP protein expression levels. Quantification results revealed that DIHL significantly increased APP expression in both the cortex and hippocampus of 5xFAD mice (*n* = 6–8). (**D**) Western blot analysis of Aβ and BACE1 protein expression levels. Quantification results indicated that DIHL significantly increased Aβ expression in both the cortex and hippocampus of 5xFAD mice, while BACE1 showed no significant change (*n* = 3). Data are presented as the mean ± SEM. Statistical significance was assessed using ANOVA with Tukey’s post hoc test, supplemented by Student’s *t*-test where appropriate. Significance levels are indicated as *P < 0.05, **P < 0.01, and ***P < 0.001

Extracellular deposition of Aβ, formed through sequential cleavage of APP in the cell membrane, is suggested to play a central role in AD pathogenesis (Chen et al. 2017). To examine the effect of HL on Aβ deposition, the accumulation of Aβ in the hippocampus and cortex was compared between WT and 5xFAD mice using immunohistochemical staining. Figure 2A shows the brain regions analyzed and illustrates the areas depicted in the representative images. Aβ predominantly accumulates in the cortex and hippocampus of the mouse brain. Figure 2B and Supplementary Figure 5 illustrate that Aβ levels are significantly higher in the cortex (WT vs. 5xFAD: 7.7 ± 1.9 vs. 100.2 ± 17.1; *p* < 0.01) and hippocampus (WT vs. 5xFAD: 15.5 ± 4.1 vs. 100.1 ± 15.2; *p* < 0.05) of 5xFAD mice than that in WT mice. Additionally, the deposition of Aβ in the cortex (5xFAD control vs. 5xFAD DIHL: 100.2 ± 17.1 vs. 176.4 ± 15.6; *p* < 0.05) and hippocampus (5xFAD control vs. 5xFAD DIHL: 100.1 ± 15.2 vs. 156 ± 14; *p* < 0.05) were significantly higher in the DIHL group than in the control group. Subsequently, APP protein expression was evaluated using Western blot analysis. Consistent with the immunohistochemical staining results demonstrating increased Aβ accumulation in the cortex and hippocampus of the DIHL group, a significant increase was observed in APP protein expression in both regions compared to that in the control group (Figure 2C). In the cortex and hippocampus of Tg2576 mice, APP protein expression was higher in the DIHL group than in the control group (Supplementary Figure 6). These findings indicate that HL increases APP expression, contributing to enhanced Aβ accumulation (Figure 2 and Supplementary Figure 6). However, given that APP expression alone may not fully represent Aβ pathology, the protein expression levels of Aβ and β-site APP cleaving enzyme 1 (BACE1) were measured using Western blot analysis. Figure 2D reveals a significant increase in Aβ levels in the 5xFAD model compared to WT, with further elevation observed in the 5xFAD DIHL group compared to the 5xFAD control group. No significant changes in BACE1 expression were observed across the groups. These findings suggest that BACE1 may not be the sole factor driving the elevated Aβ levels observed in this model. Other mechanisms, such as altered γ-secretase activity, increased Aβ peptide production via alternative pathways, or impaired Aβ clearance, may significantly contribute to Aβ accumulation in the 5xFAD HL model (Hampel et al. 2020; Sayad et al. 2022). This highlights the complexity of Aβ metabolism and its regulation through multiple enzymatic pathways, which may function independently of BACE1 activity. Additionally, the effect of HL on tau phosphorylation, a crucial event in AD pathology, was examined. Phosphorylated tau is a critical pathological marker in AD, contributing to neuronal dysfunction and the formation of NFTs (Porzig et al. 2007). While the presence of NFTs in the 5xFAD model remains debated, recent studies have demonstrated positive staining for phosphorylated tau (Ser202/Thr205) in the cortex and hippocampus of female 5xFAD mice aged 6–8 months (Selkoe 2001). To investigate the effect of HL on tau phosphorylation, both immunohistochemical staining and Western blot analysis were used. Immunohistochemical analysis using the AT8 antibody revealed increased tau phosphorylation in the cortex and hippocampus of WT and 5xFAD mice, with HL significantly increasing this effect. These findings suggest that HL may exacerbate tau-related neurodegeneration. Furthermore, Western blot analysis was used to confirm these findings, revealing an increase in tau phosphorylation at Ser202/Thr205 in the cortex and hippocampus of 5xFAD mice exposed to HL. These findings highlight the potential role of HL in exacerbating tau pathology, offering further insights into the mechanisms of neurodegeneration in AD (Supplementary Figure 7).

### Hearing impairments increase mTOR, p70S6K, and S6 phosphorylation in the brain tissue of 5xFAD mice

**Fig. 3:**
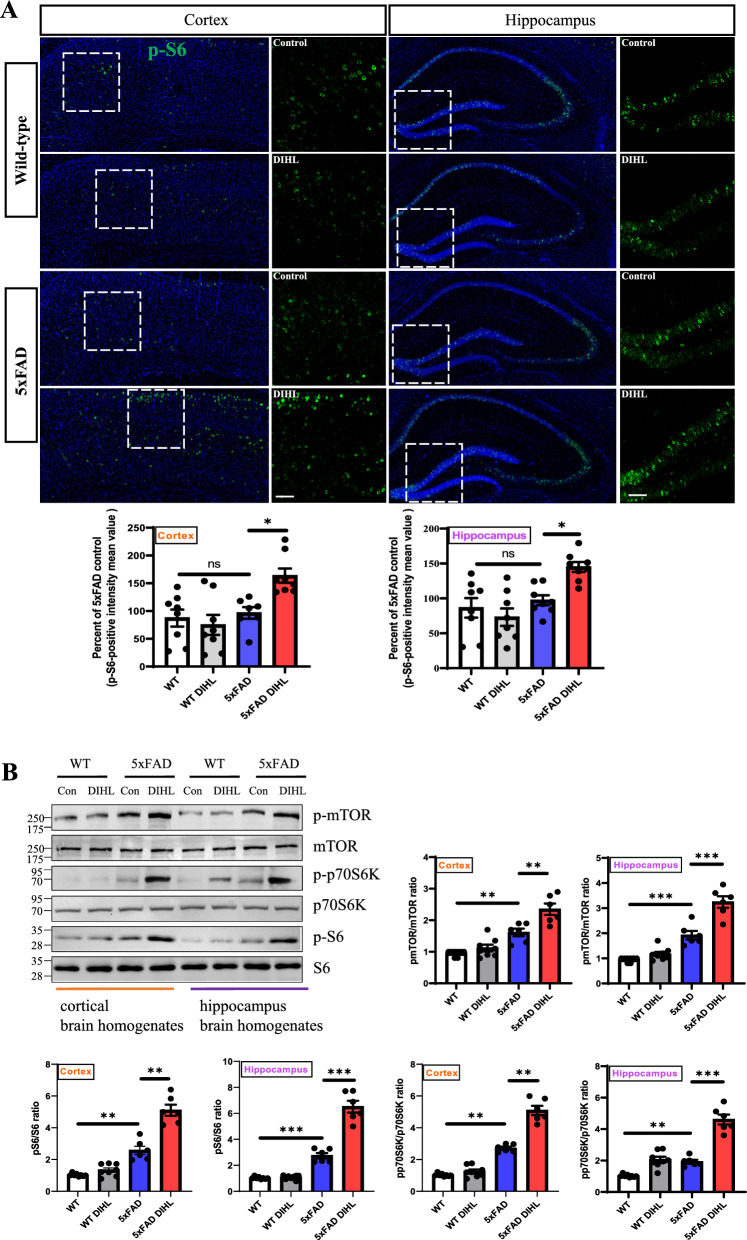
Hearing impairment increases the protein expression of the p-mTOR, p-p70S6K, and p-S6 pathways. **A** Representative immunofluorescence images show p-S6 expression in the cortex (left) and hippocampus (right) of WT and 5xFAD mice. Data are expressed as the intensity of p-S6-positive cells per 1 mm^2^ (*n* = 7–8). Scale bars represent 100 μm. **B** Western blotting was performed to analyze the protein expression levels of p-mTOR, p-p70S6K, and p-S6 (n = 6–8). Additionally, DIHL significantly increased the protein expression levels of p-mTOR, p-p70S6K, and p-S6 in both the cortex and hippocampus of 5xFAD mice. Data are presented as the mean ± SEM. Statistical significance was evaluated using ANOVA with Tukey’s post hoc test, supplemented by Student’s *t*-test where appropriate. Significance levels are indicated as *P < 0.05, **P < 0.01, and ***P < 0.001

Besides Aβ accumulation, a key marker of AD, the mTOR plays a crucial role in neurodegeneration induced by these proteins. mTOR regulates several crucial cellular processes, such as growth, metabolism, and synaptic plasticity, all of which are disrupted in AD. mTOR is involved in modulating neuroinflammation, a hallmark of AD pathology. Studies indicate that mTOR regulates microglial activation and the production of pro-inflammatory cytokines, both of which contribute to neurodegeneration (Blomberg et al. 2019; Saxton and Sabatini 2017). Activation of the mTOR pathway accelerates cellular and organismal aging, while its inhibition extends lifespan in mice (Harrison et al. 2009). Previous studies reveal that postmortem human AD brains exhibit elevated levels of phosphorylated mTOR and p70S6K. The mTOR signaling pathway, along with its downstream components—serine/threonine kinase p70S6K and ribosomal protein S6—is often activated in AD (Pei and Hugon 2008; An et al. 2003). Consequently, the phosphorylation of S6—significant indicators of AD—was assessed via immunohistochemical staining of the hippocampus and cortex in WT and 5xFAD mice. Figure 3A and Supplementary Figure 8 show that, compared to control mice, the 5xFAD DIHL group mice exhibited a significant increase in S6 phosphorylation in the cortex (5xFAD control vs. 5xFAD DIHL 100 ± 16.5 vs. 159.7 ± 10.1; *p* < 0.05) and hippocampus (5xFAD control vs. 5xFAD DIHL 100 ± 11.4 vs. 142 ± 6.1; *p* < 0.05). The mTOR signaling pathway, along with its downstream components p70S6K and S6, is crucial for regulating cellular growth and metabolism (Laplante and Sabatini 2012). Subsequently, the expression of the mTOR signaling pathway and its downstream components, including p70S6K and S6, was assessed using Western blot analysis. The phosphorylation of mTOR, p70S6K, and S6 was significantly higher in the DIHL group of 5xFAD mice than in the control group (Figure 3B). This finding indicates a strong association between HL-induced mTOR signaling activation and the progression of AD.

### Hearing impairments enhance neuroinflammation in the brain tissue of 5xFAD mice

**Fig. 4:**
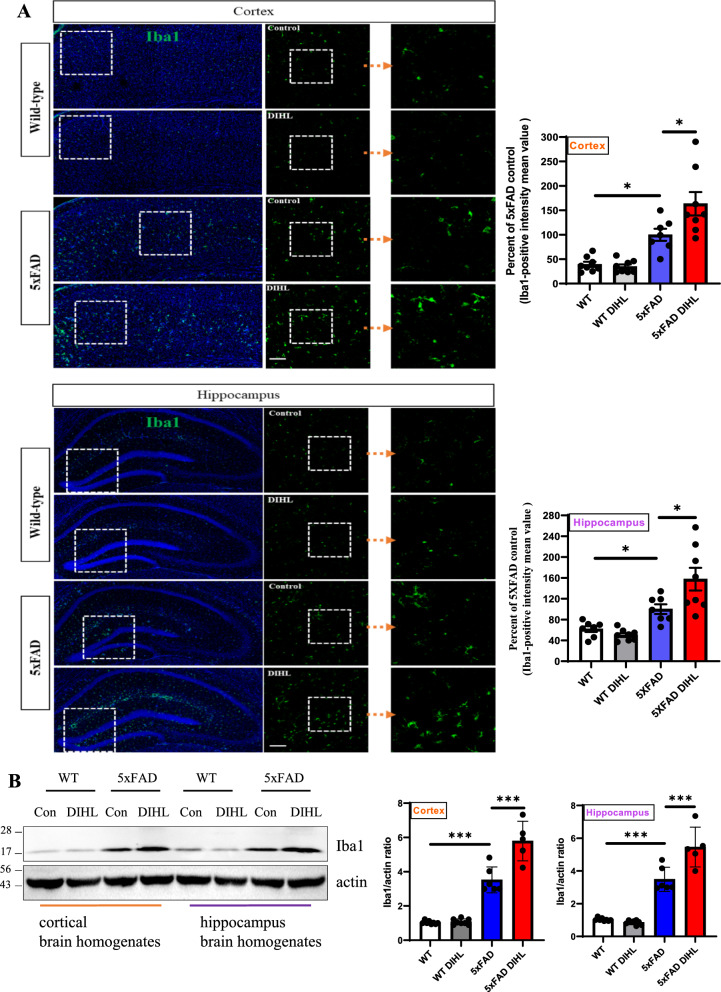
Hearing impairment also activates microglia, and DIHL increases the protein expression of Iba1. **A** Representative immunofluorescence images illustrate Iba1 expression in the cortex (upper) and hippocampus (lower) of WT and 5xFAD mice. Data are expressed as the intensity of Iba1-positive cells per 1 mm^2^ (*n* = 7–8). Scale bars represent 100 μm. **B** Western blotting was performed to analyze the protein expression levels of Iba1 (*n* = 5–8). Quantification results revealed that DIHL significantly increased Iba1 expression levels in both the cortex and hippocampus of 5xFAD mice. Data are presented as the mean ± SEM. Statistical significance was evaluated using ANOVA with Tukey’s post hoc test, supplemented by Student’s *t*-test where appropriate. Significance levels are indicated as *P < 0.05, **P < 0.01, and ***P < 0.001

**Fig. 5:**
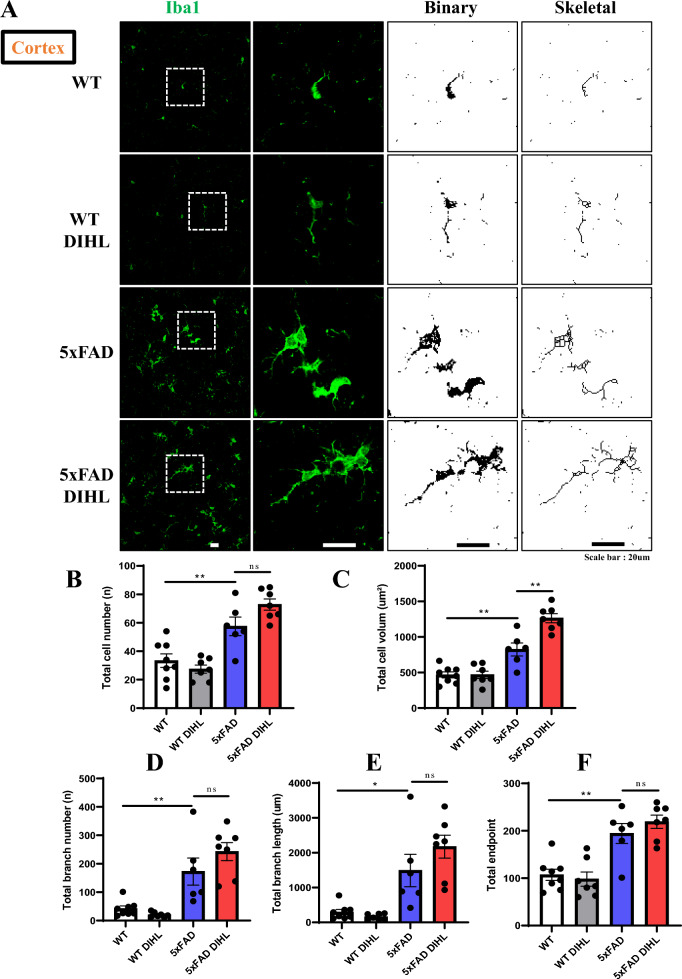

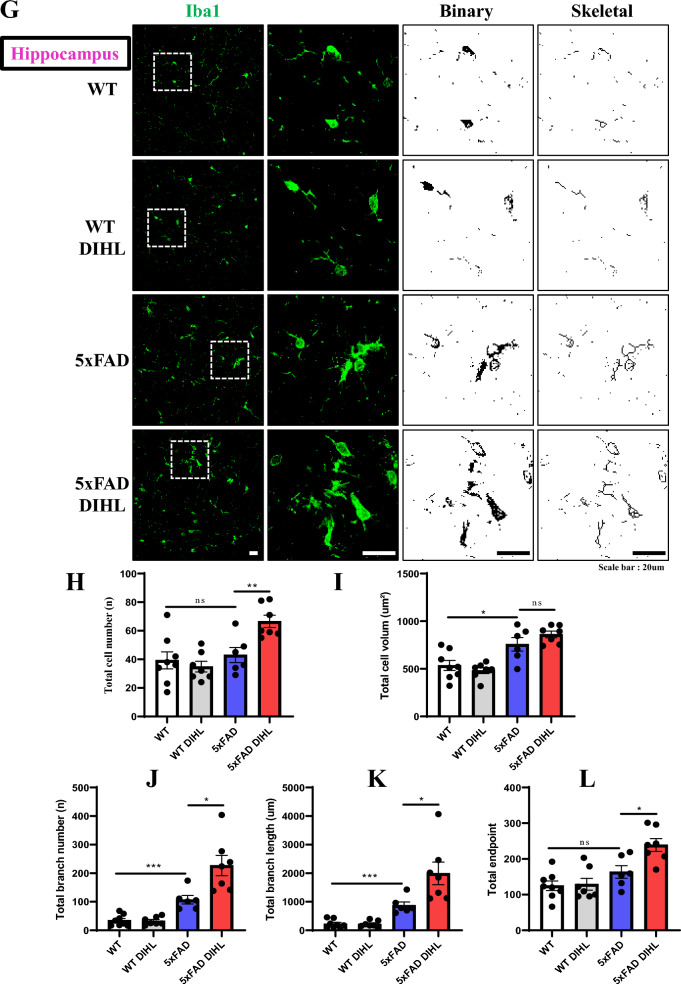
Effect of hearing impairments on microglial morphology. We performed a full photomicrograph analysis using ImageJ to assess microglial morphology. **A** Representative immunofluorescence images show photomicrographs of Iba1-stained microglia at 400 × magnification in the cortex. The Iba1 photomicrograph was converted into a binary image and subsequently transformed into a skeletal framework. Cortical analyses include the following parameters: (**B**) Microglial total cell number, (**C**) microglial total cell volume, (**D**) microglial branch number, (**E**) microglial branch length, and (**F**) microglial endpoint analysis. **G** Representative immunofluorescence images show photomicrographs of Iba1-stained microglia at 400 × magnification in the hippocampus. Hippocampal analyses include the following parameters: (**H**) Microglial total cell number, (**I**) microglial total cell volume, (**J**) microglial branch number, (**K**) microglial branch length, and (**L**) microglial endpoint analysis (n = 7–8). Scale bars represent 20 μm. Data are presented as the mean ± SEM. Statistical significance was evaluated using ANOVA with Tukey’s post hoc test, supplemented by Student’s *t*-test where appropriate. Significance levels are indicated as *P < 0.05, **P < 0.01, and ***P < 0.001

**Fig. 6:**
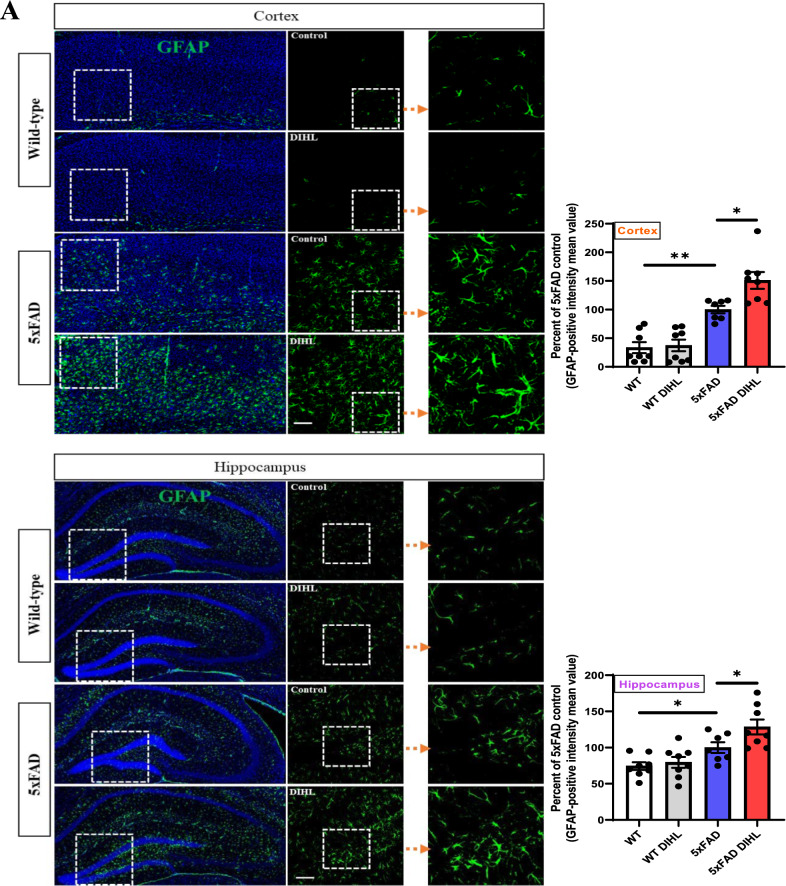

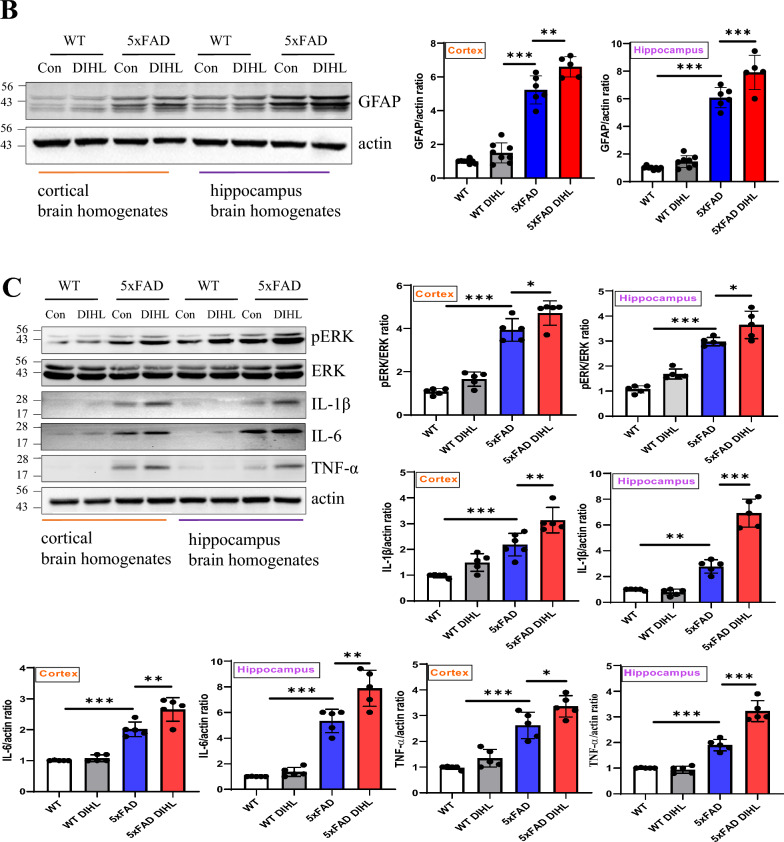
Hearing impairment activates astrocytes. Additionally, DIHL increases the protein expression of GFAP. **A** Representative immunofluorescence images of GFAP in the cortex (upper) and hippocampus (lower) of WT and 5xFAD mice are shown. Data are presented as the intensity of GFAP-positive cells per 1 mm^2^ (*n* = 7–8). Scale bars indicate 100 μm. **B** Western blotting was performed to analyze the protein expression levels of GFAP (n = 5–8). The quantification results showed that DIHL significantly increased GFAP expression in both the cortex and hippocampus of 5xFAD mice. **C** Western blotting was also used to analyze the protein expression levels of p-ERK, IL-1β, IL-6, and TNF-α (*n* = 5). The quantification results showed that DIHL significantly increased the levels of p-ERK, IL-1β, IL-6, and TNF-α in both the cortex and hippocampus of 5xFAD mice. Data are presented as the mean ± SEM. Statistical significance was evaluated using ANOVA with Tukey’s post hoc test, supplemented by Student’s *t*-test where appropriate. Significance levels are indicated as *P < 0.05, **P < 0.01, and ***P < 0.001

**Fig. 7:**
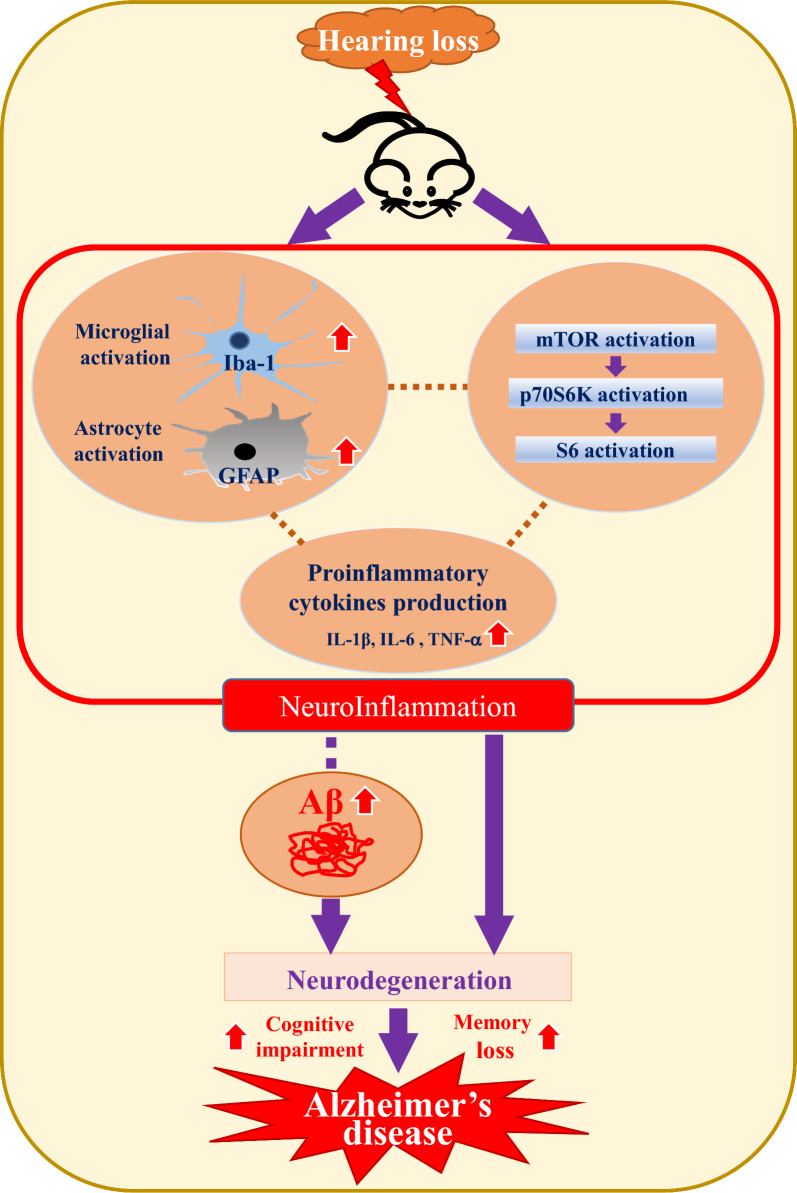
A representative schematic illustrating how hearing impairment worsens AD. Hearing impairment significantly increases the accumulation of Aβ. Additionally, it may activate the mTOR pathway, astrocytes, and microglia in the brain, contributing to increased neuroinflammation. This figure illustrates the potential associations between Aβ pathology, inflammation, and the mTOR pathway, highlighting their possible roles in AD progression

Recent studies have identified a persistent immune response in the brain as a critical third pathological feature of AD. This response involves prolonged activation of resident brain macrophages, such as microglia, along with other immune cells, such as astrocytes, which further exacerbate amyloid pathology. Prolonged pro-inflammatory cytokine signaling and neurotoxin release through microglia exacerbate neuroinflammation and neurodegeneration, further activating microglia (Lian et al. 2016; Mallach et al. 2024). This study aimed to investigate whether HL induces microglial or astrocyte activation. The levels of Iba1 and GFAP, established markers of microglial or astrocyte activation, were examined using immunostaining and Western blot analysis. In WT mice, microglia exhibited a ramified morphology, while in the 5xFAD model, microglia were activated and exhibited an amoeboid morphology. Figure 4A and Supplementary Figure 9A show that in 5xFAD mice, immunoreactive Iba1 levels were significantly higher than those in WT mice in the cortex (WT vs. 5xFAD 39.3 ± 5.4 vs. 100 ± 12.3; *p* < 0.01) and hippocampus (WT vs. 5xFAD 64.4 ± 5.5 vs. 100 ± 9.3; *p* < 0.001). Microglial activation was significantly greater in the DIHL group than in the control group, in the cortex (5xFAD control vs. 5xFAD DIHL 100 ± 12.3 vs. 163.4 ± 23.8; *p* < 0.001) and hippocampus (5xFAD control vs. 5xFAD DIHL 100 ± 9.3 vs. 157.5 ± 21.8; *p* < 0.001). To further validate these findings, Iba1 levels were assessed using Western blot analysis. Figure 4B shows that Iba1 protein expression was higher in 5xFAD mice than in WT mice. The most significant increase in the cortex and hippocampus was observed in the DIHL group of 5xFAD mice, compared to the control group. To validate the microglial profile, image processing was conducted using ImageJ to analyze microglial cell number, size, branch number, and endpoint morphology. Figure 5A–L depicts a significant increase in all assessed parameters, indicating enhanced microglial activation. DIHL induced significant microglial activation in the cortex and hippocampus of 5xFAD mice. Subsequently, the effect of hearing impairment on GFAP activation was examined through immunostaining. Figure 6A and Supplementary Figure 9B show that GFAP immunoreactivity was higher in the cortex (WT vs. 5xFAD 33.4 ± 9.5 vs. 100 ± 6.6; *p* < 0.01) and hippocampus (WT vs. 5xFAD: 71.5 ± 4.7 vs. 104.1 ± 6.6; *p* < 0.05) of 5xFAD mice than that in WT mice. Astrocyte activation, as indicated by GFAP immunoreactivity, was significantly higher in the cortex (5xFAD control vs. 5xFAD DIHL: 100 ± 6.6 vs. 145.2 ± 17.2; *p* < 0.05) and hippocampus (5xFAD control vs. 5xFAD DIHL: 104.1 ± 6.6 vs. 138.1 ± 9.5; *p* < 0.05) in the DIHL group than in the control group. To further validate these findings, GFAP expression was assessed using Western blotting, confirming the immunostaining results. GFAP protein expression increased in 5xFAD mice compared to that in WT mice, with a significant increase observed in the DIHL group compared to that in the control group (Fig. 6B). Further details on microglial signaling and cytokine involvement in AD have been previously reported (Lau et al. 2021). This study demonstrates that chronic microglial activation, driven by damage-associated molecular patterns (DAMPs) such as Aβ and hyperphosphorylated tau, induces the release of pro-inflammatory cytokines, such as TNF-α and IL-1β, which exacerbate neuroinflammation and drive the progression of AD. Additionally, the effect of HL on pro-inflammatory cytokine production was explored. The expression levels of pro-inflammatory cytokines, including IL-1β, IL-6, and TNF-α, were significantly higher in the 5xFAD DIHL group than in the control group (Fig. 6C). In the cortex and hippocampus of Tg2576 mice, Iba1 and GFAP expression were elevated in the DIHL group compared to those in the control group, as demonstrated using immunostaining and Western blot analyses (Supplementary Figure 10). These findings indicate that HL is associated with the activation of Iba1 and GFAP, resulting in increased production of pro-inflammatory cytokines. Furthermore, gliosis, characterized by the proliferation and hypertrophy of glial cells, has been identified as a contributing factor to brain dysfunction. Gliosis exacerbates tissue changes and inflammatory responses following brain damage, potentially adversely affecting neurological function (Burda and Sofroniew 2014; Sousa 2022). In summary, this study demonstrates that HL significantly increases Aβ accumulation in the brain. Activation of the mTOR pathway, astrocytes, and microglia may be associated with increased neuroinflammation (Fig. 7).

## Discussion

AD is characterized by significant neuronal loss and the deposition of abnormal proteins, primarily extracellular Aβ plaques (Selkoe 2001). The accumulation of these plaques is associated with the behavioral symptoms of AD, which is due to the damage and loss of synapses involved in memory and cognition. The Aβ cascade hypothesis suggests that Aβ aggregation initiates a cascade leading to tau pathology and neurodegeneration (Busche and Hyman 2020). Recent studies suggest that HL, particularly age-related hearing loss (ARHL), contributes to Aβ accumulation, potentially accelerating AD pathology. HL activates microglia and induces the release of pro-inflammatory cytokines, exacerbating Aβ deposition (Beckmann et al. 2020; Li et al. 2018). Additionally, HL may influence APP processing (Sirisi et al. 2024; Zheng and Koo 2006) or disrupt the blood-brain barrier (Wang et al. 2021), potentially exacerbating Aβ accumulation. This process involves tau aggregation, oxidative stress, and inflammation, which collectively leads to neuronal dysfunction and cell death. Despite ongoing efforts to reduce amyloid deposition, clinical outcomes have remained unsatisfactory, suggesting that Aβ alone may not fully drive AD pathology (Panza et al. 2019; Small and Duff 2008; Salloway et al. 2014). This suggests that Aβ alone may not fully drive AD pathology, indicating that targeting Aβ exclusively in AD lesions may not offer a comprehensive therapeutic approach.

Deficits in critical neurosensory systems, such as hearing, are well-established risk factors for AD. A consistent correlation between hearing loss and cognitive decline has been reported (Livingston et al. 2020; Lin et al. 2011b). ARHL is recognized as both a potential biomarker and a modifiable risk factor for cognitive decline (Oakley et al. 2006). Progressive HL significantly affects hippocampal synaptic plasticity, spatial memory, and neurotransmitter receptor expression (Kang et al. 2018). Owing to the rapid progression of AD in the 5xFAD mice, identifying an optimal time point for cognitive and LTP testing has proven challenging. Therefore, the Tg2576 model was used to demonstrate that HL impairs cognitive function and synaptic plasticity (Supplementary Figure 3 and 4). This finding suggests that synaptic damage plays a critical role in AD progression, further exacerbated by HL. Previous studies have connected HL to increased Aβ accumulation and brain atrophy (Ralli et al. 2019; Beckmann et al. 2020). However, the causal relationship between HL and AD remains unclear, highlighting the need for further studies to investigate the mechanisms underlying the interaction between HL and AD. These findings suggest that HL contributes to AD neuropathology by promoting neuroinflammation, synaptic damage, and Aβ accumulation. HL specifically activates microglia, triggering the release of pro-inflammatory cytokines, which can exacerbate brain inflammation (Beckmann et al. 2020). Additionally, HL may disrupt neuronal plasticity and accelerate neurodegeneration through mechanisms involving Aβ deposition and tau aggregation (Panza et al. 2019). Consistent with this, these findings demonstrate an association between HL and elevated Aβ levels in the brain of 5xFAD mice (Fig. 2). This suggests the idea that HL exacerbates AD pathology by promoting chronic inflammation and Aβ-related neurodegeneration.

Emerging evidence suggests that mTOR levels and its downstream signaling pathways, such as p70S6K in the hippocampus, are elevated. This elevation is associated with increased production of pro-inflammatory cytokines, including IL-1β, IL-6, and TNF-α, which contribute to AD pathophysiology (Wang et al. 2016; Kinney et al. 2018). mTOR regulates protein synthesis and long-term neuronal plasticity, and its dysregulation has been associated with cognitive impairments in AD (Wang et al. 2016). Consistent with these findings, mTOR and its downstream signaling pathways were upregulated in the hippocampus and cortex of 5xFAD mice compared to that in the controls. This finding suggests that mTOR signaling contributes to the pathogenesis of AD. Furthermore, these findings indicate that HL activates the mTOR pathway and its downstream signaling pathways (Fig. 3). This supports the hypothesis that mTOR signaling plays a significant role in AD progression, potentially exacerbating associated pathological changes. A previous study demonstrated that pS6 granules in the hippocampus of patients with AD are localized to intracellular structures and correlate with neurodegenerative processes (Castellani et al. 2011). Elevated pS6 levels were specifically observed in AD brains (Fig. 3). These findings highlight the activation of the mTOR pathway in AD pathology and are consistent with the results of this study, revealing that HL activates the mTOR pathway and its downstream signaling components. This activation suggests that mTOR signaling contributes to AD progression, potentially exacerbating the pathological changes associated with the disease.

Recent evidence increasingly supports the role of chronic inflammatory processes in age-related pathologies. The presence of persistent inflammation in the brains of patients with AD may be associated with neuronal and immune responses. This sustained inflammation contributes to neurodegeneration and exacerbates Aβ pathology (Li et al. 2018). An inflammatory response has been suggested as a potential link between early Aβ pathology and subsequent NFT development. Various studies indicate that inflammation serves as a central mechanism driving Aβ pathology and its progression (Li et al. 2018; Wang et al. 2011). Furthermore, HL, including NIHL, has been shown to increase the expression of pro-inflammatory cytokines and activate microglia, resulting in neuroinflammation. This process disrupts synaptic balance and contributes to conditions such as tinnitus (Xie et al. 2021; Wang et al. 2019). While inflammation is recognized as a critical area for future studies on HL, its role in the aging cochlea and central auditory pathways in the brain remains poorly understood. Therefore, targeting neuroinflammation may represent a potential therapeutic approach for treating HL-related disorders, such as AD. In this study, a significant increase in the protein expression of neuroinflammatory markers, including Iba1, GFAP, and pro-inflammatory cytokines, was observed. Emerging evidence suggests that HL may exacerbate AD progression by promoting inflammation (Seicol et al. 2022; Azeem et al. 2023). Chronic inflammation increases pro-inflammatory cytokine levels, contributing to neurodegeneration and Aβ accumulation. This finding suggests that HL could exacerbate AD pathology through its inflammatory effects. HL leads to microglial activation and the release of pro-inflammatory cytokines, exacerbating neuroinflammation. This chronic inflammatory response contributes to Aβ plaque accumulation, disrupts synaptic function, and promotes neurodegeneration. Consequently, HL may accelerate cognitive decline through immune-mediated and synaptic mechanisms. Figures 4 and 5 demonstrate that HL induces microglia activation, while Fig. 6A–B indicate the activation of astrocytes, suggesting that HL promotes neuroinflammatory responses in the brain. These findings support the hypothesis that HL may exacerbate AD progression by enhancing neuroinflammation (Li et al. 2018; Xie et al. 2021). Understanding these inflammatory mechanisms may offer valuable insights for developing therapeutic strategies to mitigate the effect of HL on AD progression.

The Aβ cascade hypothesis, widely considered the most compelling model for explaining AD pathogenesis, suggests that Aβ accumulation leads to synaptic and neuronal dysfunction (Zheng et al. 2022). While systemic inflammation may precede Aβ accumulation, it remains unclear whether the accumulation of Aβ plaques during aging is primarily due to inflammatory processes or other age-related mechanisms. Additionally, chronic and sustained stimulation can activate microglia, leading to their priming. Once primed, microglia may trigger Aβ production and promote neuroinflammation. These processes ultimately contribute to the loss of the number and function of neurons, which is strongly associated with AD. A combination of factors, including inflammation and microglial priming, plays a critical role in the onset and progression of AD (Karran and Strooper 2022). Therefore, targeting inflammatory mechanisms offers a promising approach for the therapeutic development of AD.

## Conclusions

The findings demonstrate that HL significantly increases Aβ accumulation in the brain and triggers neuroinflammation through the activation of astrocytes and microglia, accelerating AD progression. Clinical strategies targeting neuroinflammation or the mTOR signaling cascade may provide promising therapeutic approaches for treating cognitive decline and AD progression in individuals with HL.

## Supplementary Information

**Supplemetary Material 1. Figure S1. Auditory threshold evaluations of 5xFAD and Tg2576 mice**. The ABR thresholds were measured for click and tone bursts in (A) 5xFAD and (B) Tg2576 mice. **Figure S2. Establishment of a DIHL model induced by a combination of kanamycin and furosemide injections in Tg2576 mice.** (A) Experimental timeline for Tg2576 mice. (B) The ABR thresholds were measured for click and tone burst stimuli in Tg2576 mice (*n* = 6–8). Data are presented as the mean ± SEM. Statistical significance was evaluated using ANOVA with Tukey’s post hoc test, supplemented by Student’s t-test where appropriate. Significance levels are indicated as *P < 0.05, **P < 0.01, and ***P < 0.001. **Figure S3. Hearing impairment exacerbates cognitive dysfunction in both WT and Tg2576 mice.** (A) Experimental timeline of Tg2576 mice. (B) Novel object recognition performance was assessed by calculating object preference using the formula: [100 (TNO-TFO)/(TNO + TFO)] in both the WT and Tg2576 groups. T - contact time; FO - familiar object; NO - novel object. Learning and memory deficits in the DIHL group of WT and Tg2576 mice were examined. In the Y-maze test: (C) The DIHL group exhibited reduced spontaneous alternation performance (SAP). (D) The DIHL group showed increased same-arm return (SAR), indicating memory impairment (*n* = 5). Data are presented as the mean ± SEM. Statistical significance was evaluated using ANOVA with Tukey’s post hoc test, supplemented by Student’s *t*-test where appropriate. Significance levels are indicated as *P < 0.05, **P < 0.01, and ***P < 0.001. **Figure S4. Hearing impairment reduces LTP in both WT and Tg2576 mice**. (A) The experimental timeline for Tg2576 mice. (B) Brain slices were collected at either 4.5 months or 5.5 months (*n* = 5) for the LTP test. Compared with WT mice, Tg2576 mice exhibited reduced LTP. Both the WT and Tg2576 DIHL groups showed a significant decrease in LTP. Data are presented as the mean ± SEM. Statistical significance was evaluated using ANOVA with Tukey’s post hoc test, supplemented by Student’s *t*-test where appropriate. Significance levels are indicated as *P < 0.05, **P < 0.01, and ***P < 0.001. **Figure S5. Localization of the cortex and hippocampus regions for immunohistochemical staining of Aβ plaque**. The regions of the cortex and hippocampus were delineated in the Aβ plaque-stained images. **Figure S6. Hearing impairment results in a significant increase in APP expression in the brain tissue of Tg2576 mice**. Western blotting was performed to analyze APP protein expression levels. DIHL significantly increased APP expression in both the cortex and hippocampus of Tg2576 mice. Data are presented as the mean ± SEM. Statistical significance was evaluated using ANOVA with Tukey’s post hoc test, supplemented by Student’s *t*-test where appropriate. Significance levels are indicated as *P < 0.05, **P < 0.01, and ***P < 0.001. **Figure S7. Hearing impairments increased tau phosphorylation in the brain tissue of 5xFAD mice**. (A) Representative immunofluorescence images show p-tau (AT8) in the cortex (left) and hippocampus (right) of WT and 5xFAD mice. Data are presented as the intensity of p-tau (pThr205/pSer202)-positive cells per 1 mm² (*n* = 7–8). Scale bars represent 100 μm. (B) Western blotting was performed to analyze p-tau (AT8) protein expression levels. Quantitative analysis demonstrated that DIHL significantly elevated p-tau (AT8) expression levels in both the cortex and hippocampus of 5xFAD mice (*n* = 6–8). Data are presented as the mean ± SEM. Statistical significance was evaluated using ANOVA with Tukey’s post hoc test, supplemented by Student’s *t*-test where appropriate. Significance levels are indicated as *P < 0.05, **P < 0.01, and ***P < 0.001. **Figure S8. Localization of the cortex and hippocampus regions for immunohistochemical staining for S6 phosphorylation**. The regions of the cortex and hippocampus were delineated on the p-S6-stained images. **Figure S9. Localization of the cortex and hippocampus for immunohistochemical staining of Iba1 and GFAP**. The regions of the cortex and hippocampus were delineated on the (A) Iba1- and (B) GFAP-stained images. **Figure S10. Hearing impairment also activates microglia and astrocytes in the brain tissue of Tg2576 mice**. DIHL increases the protein expression levels of Iba1 and GFAP. Representative immunofluorescence images show (A) Iba1 and (B) GFAP in the cortex (left) and hippocampus (right) of WT and Tg2576 western blotting was performed to analyze the protein expression levels of GFAP and Iba1.

## Data Availability

The raw data supporting the conclusions of this article will be made available by the authors without reservation.

## Abbreviations

ABR: Auditory brainstem response
Aβ: Beta-amyloid
AD: Alzheimer’s disease
AKT: Protein kinase b
APP: Amyloid precursor protein
BACE1: β-site APP cleaving enzyme 1
DIHL: Drug-induced hearing loss
GBD: Global Burden of Disease
GFAP: Glial fibrillary acidic protein
Iba1: Ionized calcium-binding adaptor molecule 1
IL-1β: Interleukin-1β
HL: Hearing loss
LTP: Long-term potentiation
mTOR: Mammalian target of rapamycin
p70S6K: P70 ribosomal S6 kinase
NFTs: Neurofibrillary tangles
PI3K: Phosphatidylinositol 3 kinase
ROS: Reactive oxygen species
RNS: Reactive nitrogen species
TNF-α: Tumor necrosis factor-α
YLD: Years lived with disability
