# Supplementary figures and images for: Hearing modulation affects Alzheimer’s disease progression linked to brain inflammation: a study in mouse models
Source: Mol Med. 2024 Dec 26;30:276. doi: 10.1186/s10020-024-01040-1 (PMC11670416; doi:10.1186/s10020-024-01040-1)

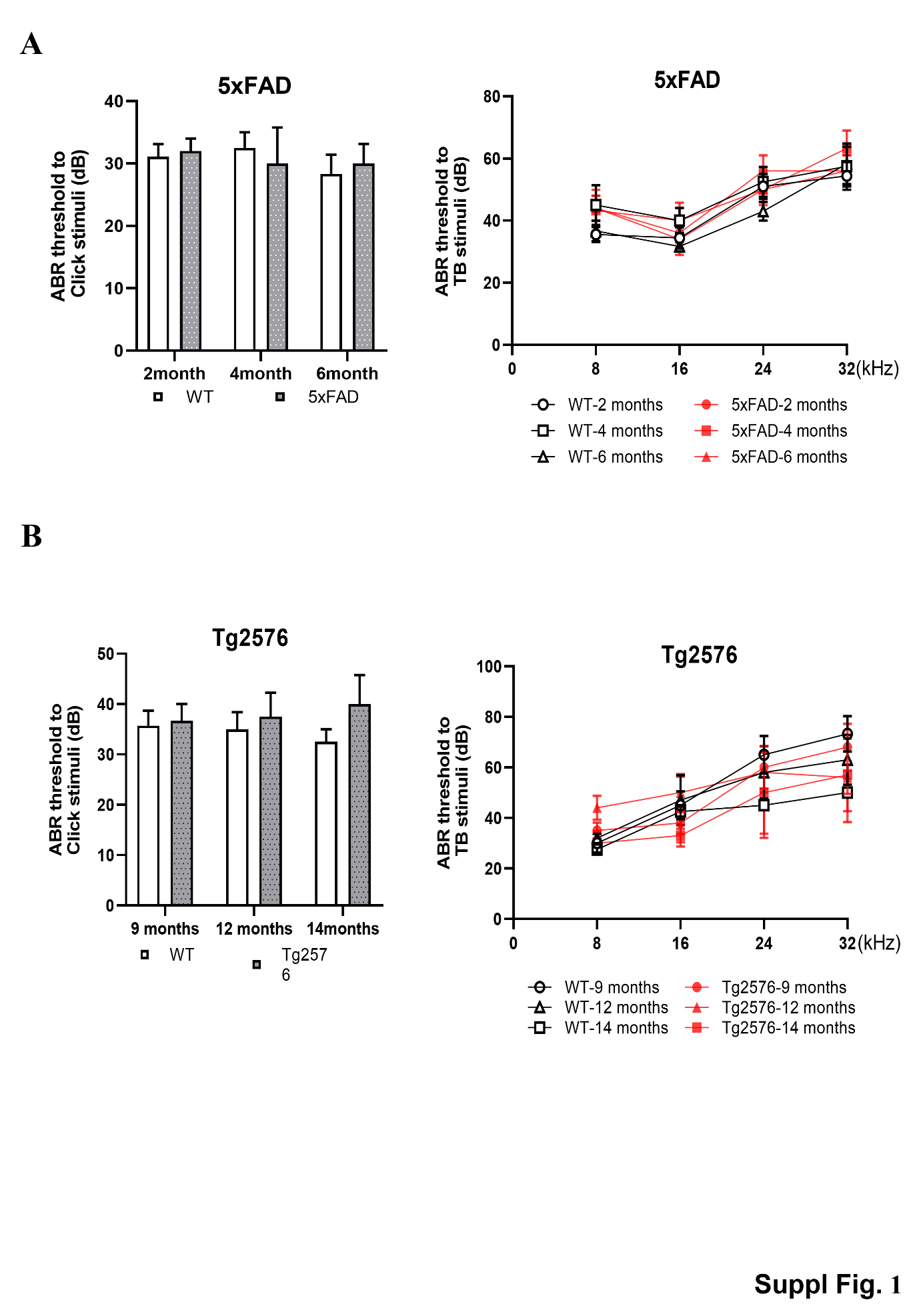

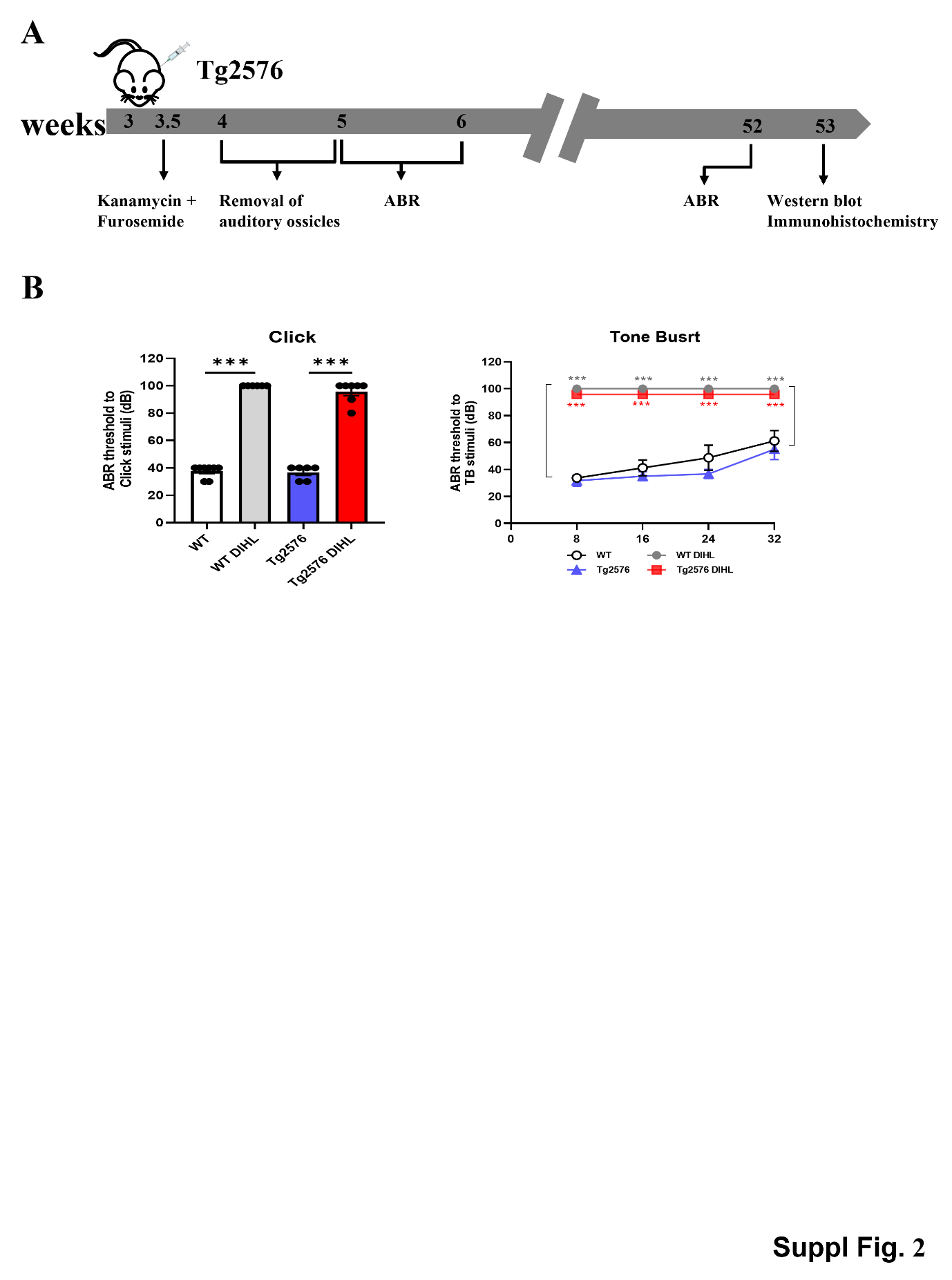


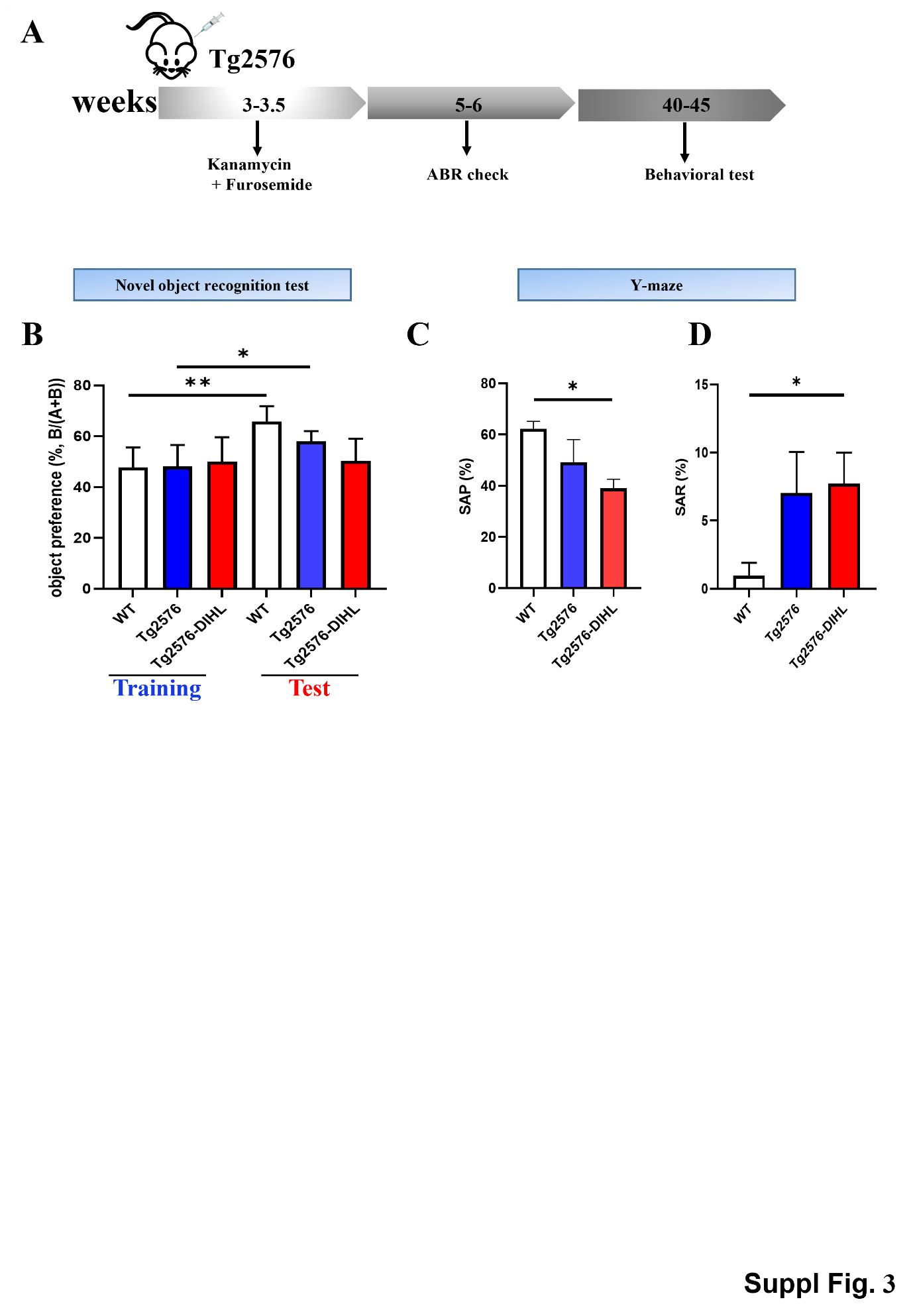

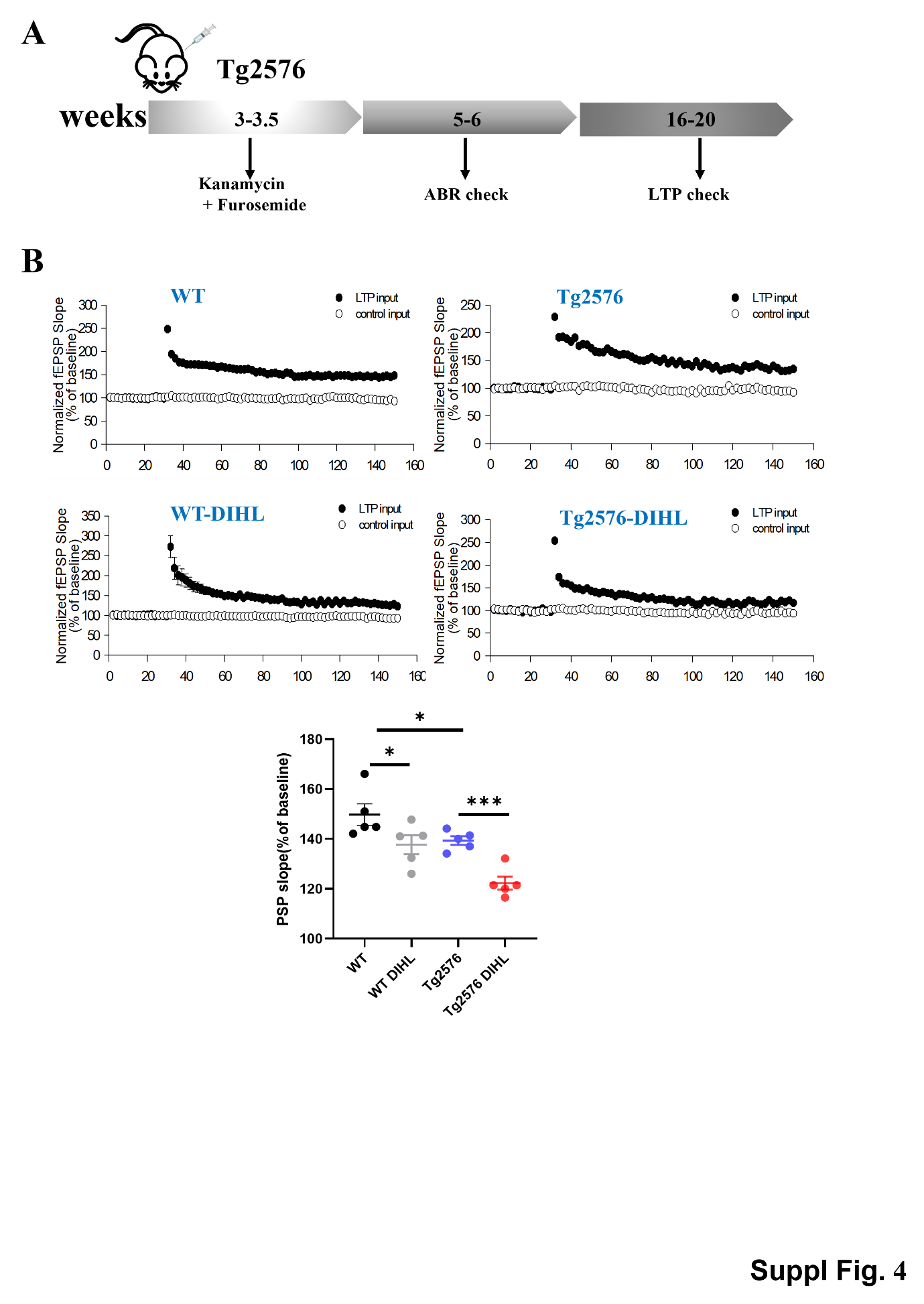

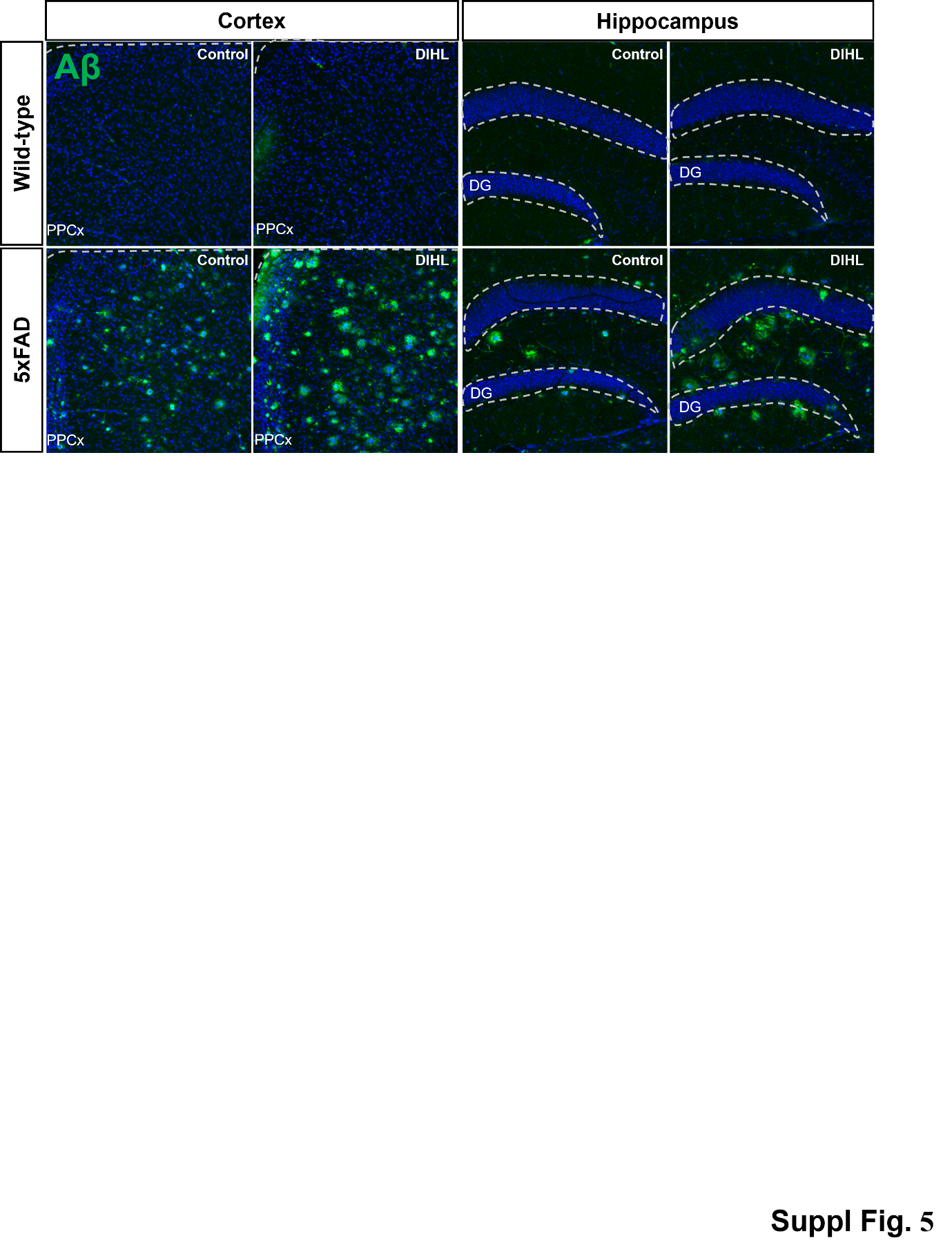

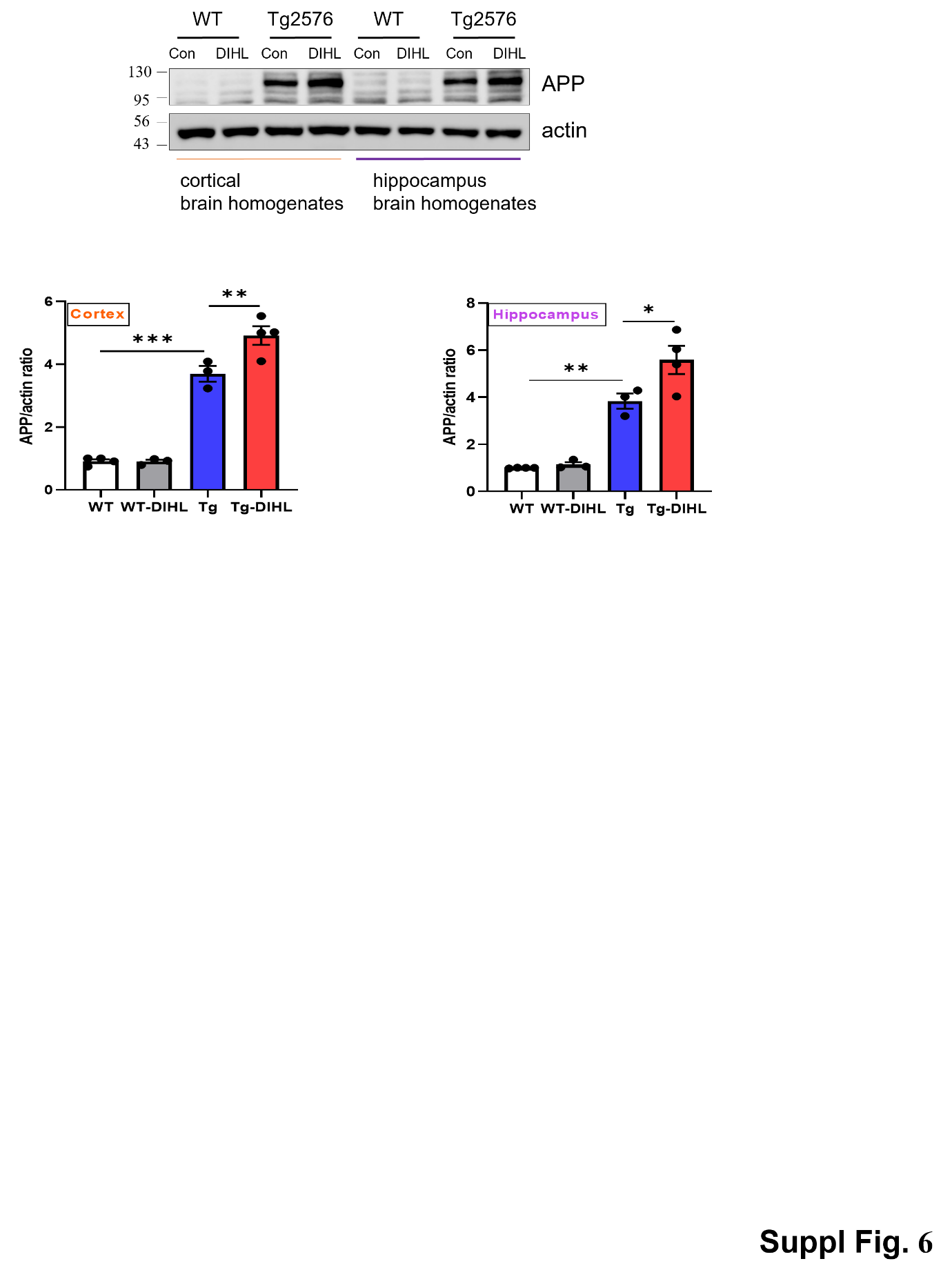

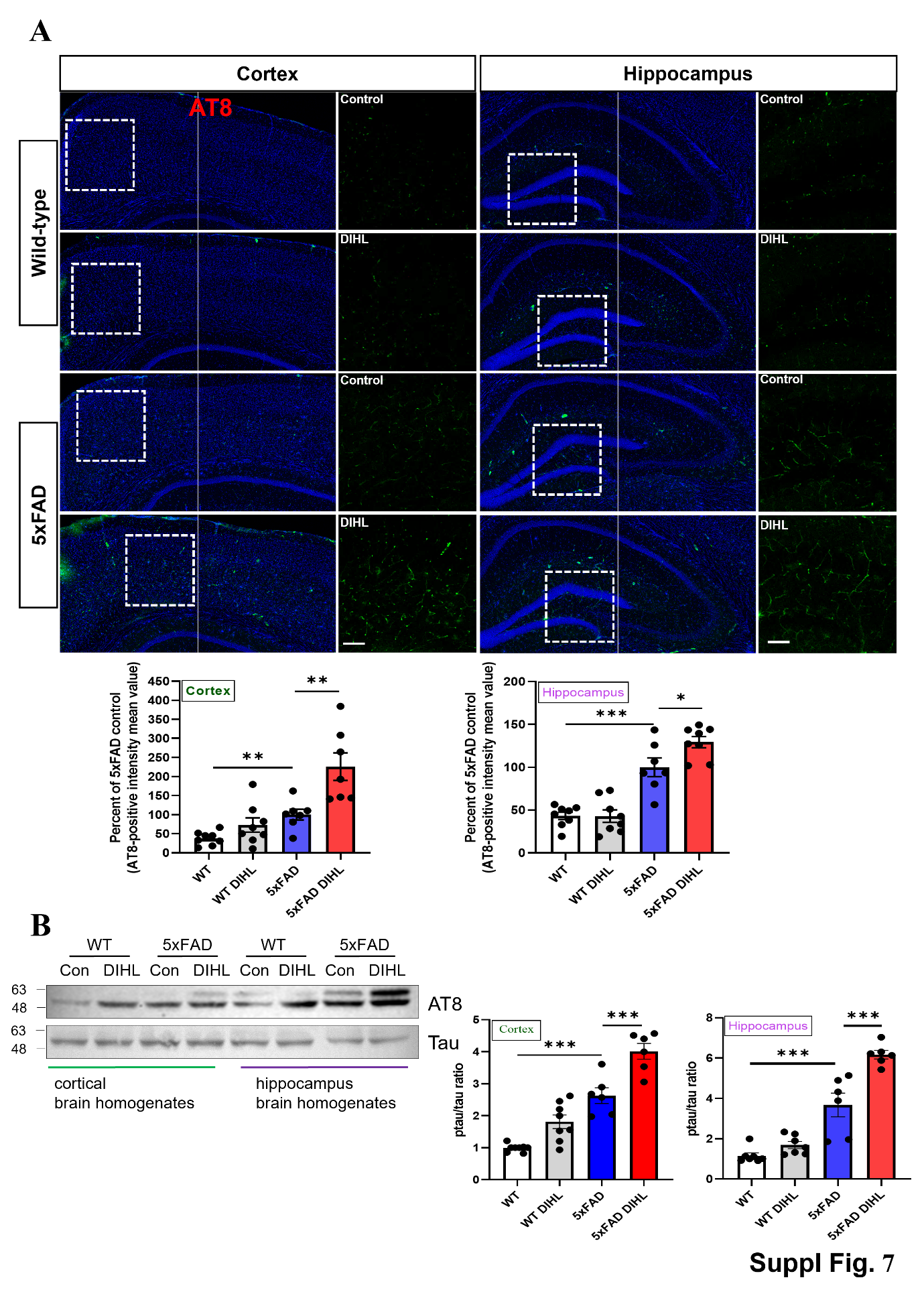

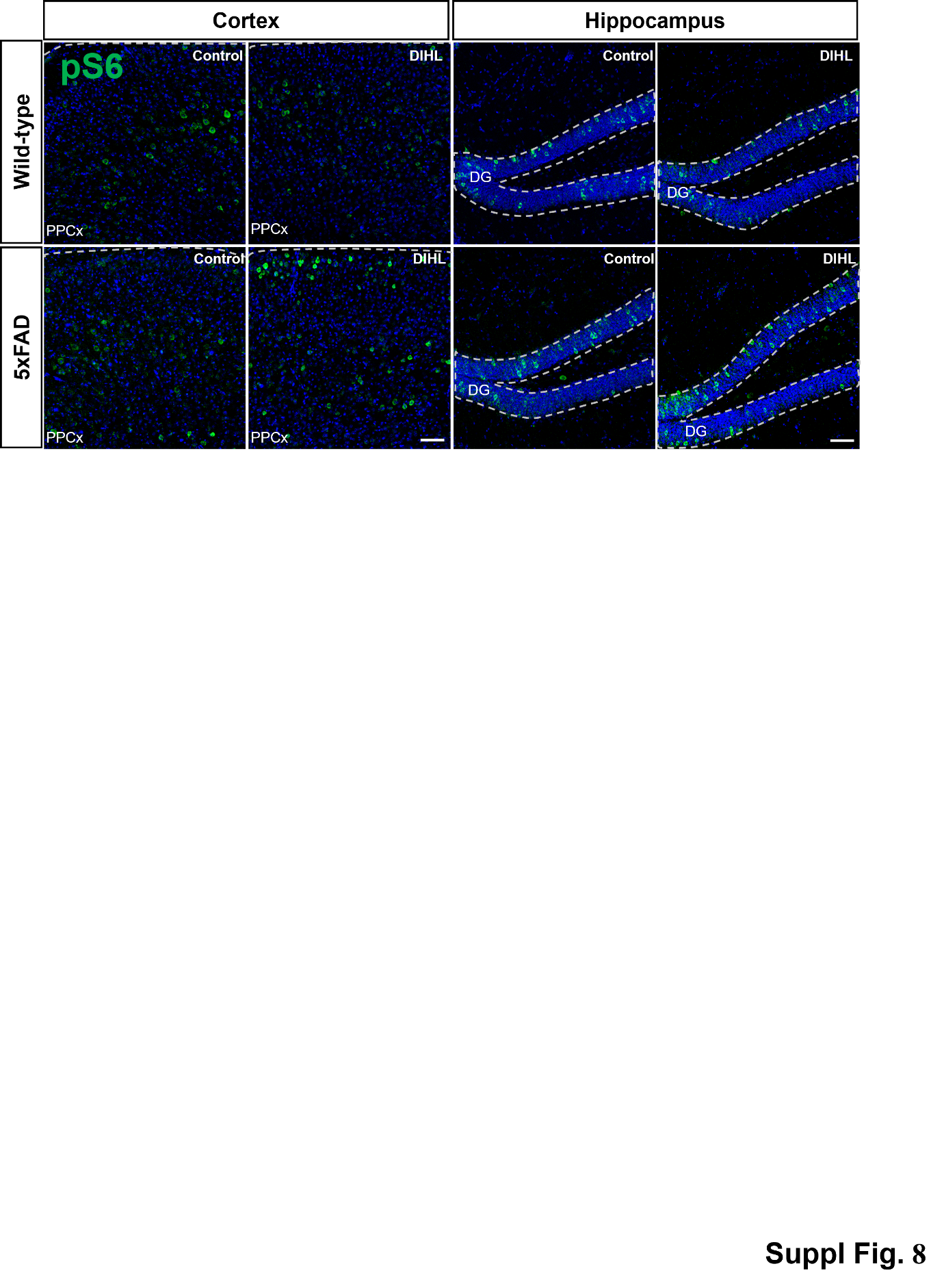

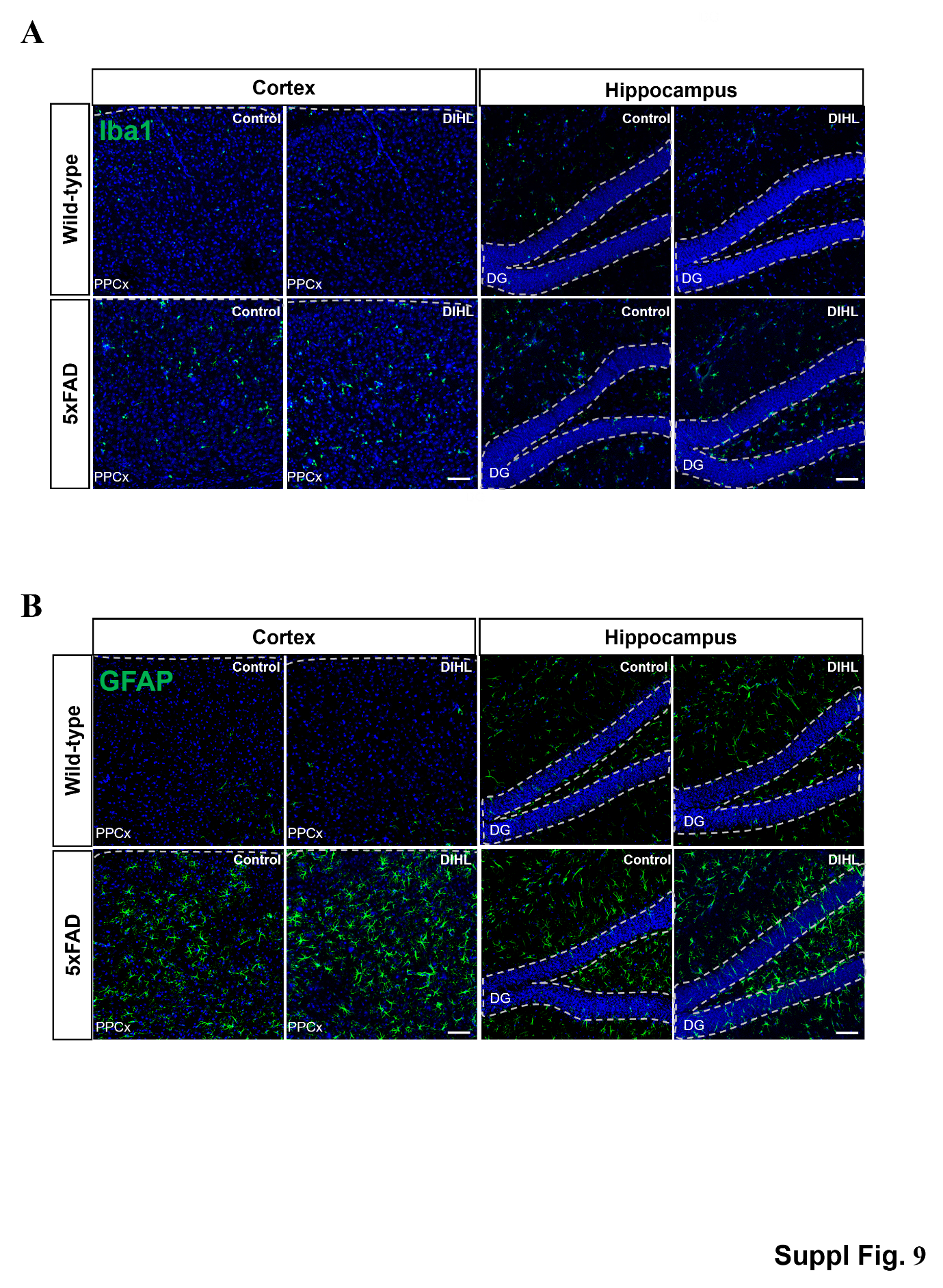

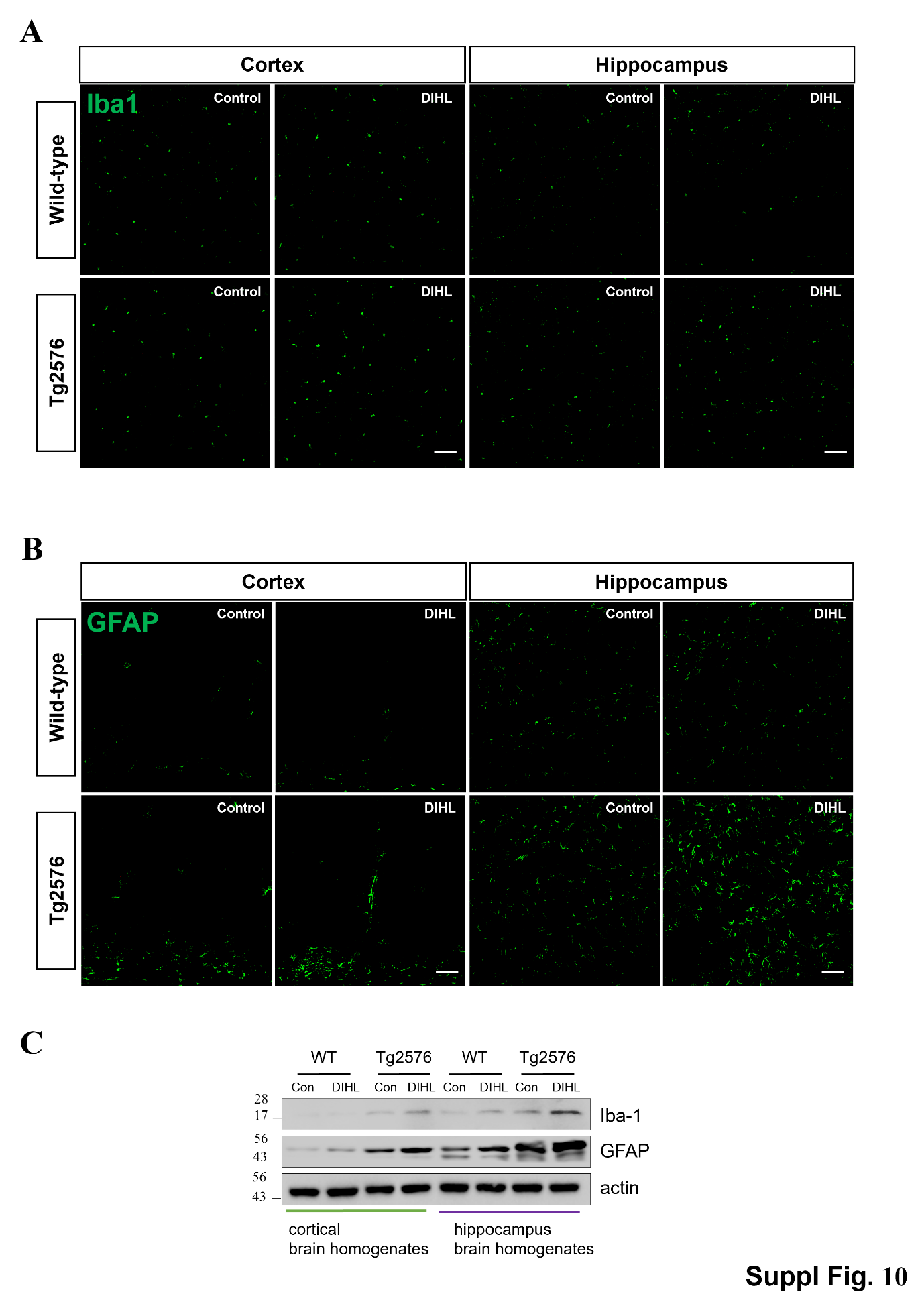

Supplement: Supplementary file 1 — Supplemetary Material 1. Figure S1. Auditory threshold evaluations of 5xFAD and Tg2576 mice. The ABR thresholds were measured for click and tone bursts in (A) 5xFAD and (B) Tg2576 mice. Figure S2. Establishment of a DIHL model induced by a combination of kanamycin and furosemide injections in Tg2576 mice. (A) Experimental timeline for Tg2576 mice. (B) The ABR thresholds were measured for click and tone burst stimuli in Tg2576 mice (n = 6–8). Data are presented as the mean ± SEM. Statistical significance was evaluated using ANOVA with Tukey’s post hoc test, supplemented by Student’s t-test where appropriate. Significance levels are indicated as *P < 0.05, **P < 0.01, and ***P < 0.001. Figure S3. Hearing impairment exacerbates cognitive dysfunction in both WT and Tg2576 mice. (A) Experimental timeline of Tg2576 mice. (B) Novel object recognition performance was assessed by calculating object preference using the formula: [100 (TNO-TFO)/(TNO + TFO)] in both the WT and Tg2576 groups. T - contact time; FO - familiar object; NO - novel object. Learning and memory deficits in the DIHL group of WT and Tg2576 mice were examined. In the Y-maze test: (C) The DIHL group exhibited reduced spontaneous alternation performance (SAP). (D) The DIHL group showed increased same-arm return (SAR), indicating memory impairment (n = 5). Data are presented as the mean ± SEM. Statistical significance was evaluated using ANOVA with Tukey’s post hoc test, supplemented by Student’s t-test where appropriate. Significance levels are indicated as *P < 0.05, **P < 0.01, and ***P < 0.001. Figure S4. Hearing impairment reduces LTP in both WT and Tg2576 mice. (A) The experimental timeline for Tg2576 mice. (B) Brain slices were collected at either 4.5 months or 5.5 months (n = 5) for the LTP test. Compared with WT mice, Tg2576 mice exhibited reduced LTP. Both the WT and Tg2576 DIHL groups showed a significant decrease in LTP. Data are presented as the mean ± SEM. Statistical significance was evaluated [file 10020_2024_1040_MOESM1_ESM.docx]
